# Climate change threatens native potential agroforestry plant species in Brazil

**DOI:** 10.1038/s41598-022-06234-3

**Published:** 2022-02-10

**Authors:** Valdeir Pereira Lima, Renato Augusto Ferreira de Lima, Fernando Joner, Ilyas Siddique, Niels Raes, Hans ter Steege

**Affiliations:** 1 Programa de Pós-Graduação em Recursos Genéticos Vegetais, Universidade Federal de Santa Catarina, Florianópolis, SC 88034-000 Brazil; 2 Departamento de Fitotecnia, Universidade Federal de Santa Catarina, Florianópolis, SC 88034-000 Brazil; 3 Naturalis Biodiversity Center, PO Box 9517, 2300 RA Leiden, The Netherlands; 4 Departamento de Ecologia, Instituto de Biociências, Universidade de São Paulo, São Paulo, SP 05508-090 Brazil; 5 NLBIF – Netherlands Biodiversity Information Facility, Leiden, The Netherlands; 6 Systems Ecology, Free University, De Boelelaan 1087, 1081 HV Amsterdam, The Netherlands

**Keywords:** Ecology, Environmental sciences

## Abstract

Climate change is one of the main drivers of species extinction in the twentyfirst-century. Here, we  (1) quantify potential changes in species' bioclimatic area of habitat (BAH) of 135 native potential agroforestry species from the Brazilian flora, using two different climate change scenarios (SSP2-4.5 and SSP5-8.5) and dispersal scenarios, where species have no ability to disperse and reach new areas (non-dispersal) and where species can migrate within the estimated BAH (full dispersal) for 2041–2060 and 2061–2080. We then (2) assess the preliminary conservation status of each species based on IUCN criteria. Current and future potential habitats for species were predicted using MaxEnt, a machine-learning algorithm used to estimate species' probability distribution. Future climate is predicted to trigger a mean decline in BAH between 38.5–56.3% under the non-dispersal scenario and between 22.3–41.9% under the full dispersal scenario for 135 native potential agroforestry species. Additionally, we found that only 4.3% of the studied species could be threatened under the IUCN Red List criteria B1 and B2. However, when considering the predicted quantitative habitat loss due to climate change (A3c criterion) the percentages increased between 68.8–84.4% under the non-dispersal scenario and between 40.7–64.4% under the full dispersal scenario. To lessen such threats, we argue that encouraging the use of these species in rural and peri-urban agroecosystems are promising, complementary strategies for their long-term conservation.

## Introduction

Hundreds of species are unquestionably promising for future human welfare^1^, yet a large number of these species may potentially be threatened by impacts of climate change in the coming decades. Climate change is one of the important drivers affecting species survival, causing global biodiversity loss^2–5^. Climate change affects species in different ways, such as altering the suitability of current habitat of species, resulting in accelerated extinction rates^2,6,7^. The United Nations Intergovernmental Panel on Climate Change (IPCC) estimates that if Earth's average temperature rises between 2 and 3 °C, about 20 to 30% of all terrestrial biodiversity will be at high risk of extinction by the end of the century^8^. In the last century, land and ocean temperature showed a warming of approximately 1.0 °C ^3^, which may increase another 1.4 °C to 5.0 °C by 2100, if we do not reduce greenhouse gas emissions^9^. The latest IPCC special report reinforces the importance of keeping the temperature increase below 1.5 °C, in order to keep negative effects on natural resources, ecosystem functioning, food security and biodiversity to a minimum^10^. Considering the potential climate change scenarios with additional temperature increases, both widespread species and narrow-ranged endemic species will likely suffer irreparable consequences with regard to their distribution range and abundance^5^.

Brazil is the world’s most biodiversity-rich country, with 33,161 known species of vascular plants^11^, and harboring some of the largest remnants of tropical old-growth forests^12^. Despite the large number of native plant species, many of which with major untapped socioeconomic potential, the Brazilian agricultural industry exploits only a few, and largely, exotic crops^13^. Agroforestry species are those that have the function of simultaneously benefiting people's livelihoods and the ecological systems while showing great potential for multi-species intercropping^14^. These species are often characterized by their multiple uses, different harvest seasons and potential for market adoption^15–18^. Several useful native Brazilian plant species are potentially suitable for pasture production, silviculture, orchards, bioenergy, green manuring, as well as in integrated, biodiverse, multifunctional agroforestry^13,19^. Besides enhancing biodiversity and promoting the socio-economic development of local communities^20–23^, agroforestry systems can play a pivotal role in mitigating the effects of climate change: they sequester more atmospheric carbon than conventional farming^24–26^. Although agroforestry practices can ameliorate the impacts of climate change in Brazil, these agroecological systems are also vulnerable^27^. Considering the rapidly increasing human demand for plant products, native plant species from megadiverse countries undoubtedly represent a reservoir of genetic diversity, providing beneficial alleles for crop improvement and higher adaptive potential to face global changes^13,28^. Changes in land use may not be the main driver impacting these species as they are widely distributed among the neotropics and are easily found along streets and city squares across Brazil, some almost ruderal, regenerating in open areas of cities^13,29^. The future impact of climate change on species distributions should be taken into account for setting conservation priorities, as well as for promoting species conservation through their sustainable use^30^.

Spatial and temporal changes of species’ suitable habitat can be predicted with ecological niche models (ENMs), the most widely used tool to assess species vulnerability to changing climatic conditions^36–38^. Besides that, modeled habitats based on climatic variables allow us to consider the impacts of climate change on the species’ area of habitat, which is the habitat available to a particular species within its range^39^. Here, to consider those impacts, we modeled the species' bioclimatic area of habitat (BAH). Species dispersal is pivotal to the survival of species in the face of rapid climate change^40^. Thus, to better understand species responses, this central process that determines the potential spread of a population needs to be addressed in conservation assessments^7^. Although several studies have sought to better understand the impact of climate change on the distribution of plant species with narrow-ranged distribution or threatened with extinction, we note that no study has yet focused on species of agroforestry interest in Brazil, which generally have widespread distribution. These species are promising for conservation-by-use, an approach used by people communities for millennia in different ecosystems in Brazil^41,42^.

Here, we apply an ENM approach to (1) quantify potential changes in BAH of 135 native potential agroforestry species from the Brazilian flora using two climate change scenarios (SSP2-4.5 and SSP5-8.5) and two dispersal (non-dispersal and full dispersal) scenarios for 2041–2060 and 2061–2080. We then (2) assess the preliminary conservation status of each species using IUCN Red List of Threatened Species criteria^43^.

## Results

### Model performance

We evaluated the model performance through a null-model for significance testing of presence-only ENMs and retained 135 significant ENMs, corresponding to 97.1% of all species. Overall, final models showed high accuracy, indicated by AUC values ranging from 0.850 ± 0.139 to 0.985 ± 0.058, demonstrating a clear ability to distinguish suitable from unsuitable habitats. We detected no spurious correlations through inspection of species-response-curves.

### Impacts on species BAH

**Figure 1:**
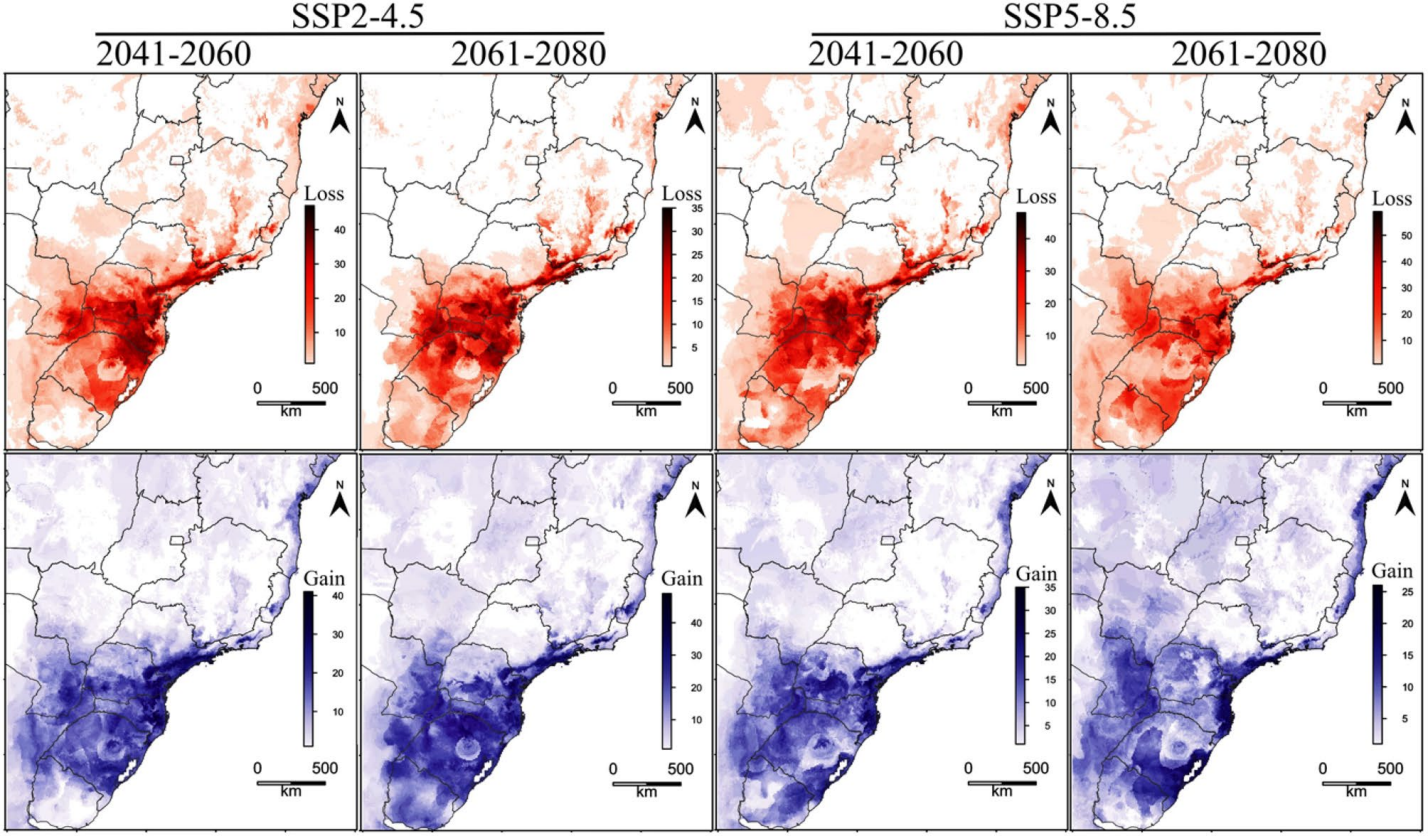
**L**oss (red) and gain (blue) of BAH for 135 native potential agroforestry plant species in Brazil, obtained by stacking ENM binary predictions, based on different climate scenarios and years. Legend indicates the number of species. Maps created with custom R script. Version R 4.1.1 (https://www.R-project.org/). Base map source (Brazilian states shapefile) obtained from the Brazilian Institute of Geography and Statistics (https://www.ibge.gov.br/).

Under the non-dispersal scenario, the average decline in BAH was predicted to be between 38.5% (SSP2-4.5) and 43.5% SSP5-8.5 by 2041–2060 and between 43.4% (SSP2-4.5) and 56.3% (SSP5-8.5) by 2061–2080. For the full dispersal scenario, however, the average decline of BAH was predicted to be between 22.3% (SSP2-4.5) and 29.7% (SSP5-8.5) by 2041–2060 and between 27.4% (SSP2-4.5) and 41.9% (SSP5-8.5) by 2061–2080 (Supplementary Table S1). Although the majority of species predicted BAH losses over different scenarios, some are predicted to experience BAH gains (Fig. 1., Table 1, Supplementary Table S1). We noticed that some species were predicted to lose their entire BAH by 2041–2060 and 2061–2080, such as the medicinal species *Cunila microcephala* in the non-dispersal scenario and the forage species *Ornithopus micranthus*, in all scenarios, except for the SSP5-8.5 (2061–2080) in the non-dispersal scenario and SSP2-4.5 and SSP5-8.5 (2061–2080) in the full dispersal scenario. Species with the greatest increase in BAH were the forage species *Indigofera sabulicola* with an increase of 388%, the ornamental species *Epidendrum fulgens* (263%), and the forage species *Echinochloa polystachya* (259%) in the SSP5-8.5 for 2061–2080 considering the full dispersal scenario (Table 1, Supplementary Table S1).

**Table 1:** Future range changes for 135 native potential agroforestry plant species in Brazil under two dispersal scenarios.

Aromatic species	NON-DISPERSAL				FULL DISPERSAL			
	2041–2060		2061–2080		2041–2060		2061–2080	
	SSP2-4.5	SSP5-8.5	SSP2-4.5	SSP5-8.5	SSP2-4.5	SSP5-8.5	SSP2-4.5	SSP5-8.5
*Capsicum flexuosum*	− 36.1	− 47.7	− 39.2	− 60.1	− 33.7	− 35.8	− 38.0	− 57.1
*Pimenta pseudocaryophyllus*	− 45.3	− 51.7	− 52.9	− 77.6	− 38.9	− 47.6	− 44.8	− 76.5
*Schinus terebinthifolia*	− 28.6	− 33.6	− 30.6	− 47.0	− 12.7	− 14.7	− 16.6	− 31.3
*Tropaeolum pentaphyllum*	− 30.9	− 29.9	− 34.1	− 39.2	− 2.9	3.1	0.1	− 14.1
**Fibrous species**								
*Coleataenia prionitis*	− 17.9	− 38.5	− 33.6	− 52.1	50.6	26.1	54.4	32.7
*Geonoma gamiova*	− 6.1	− 6.6	− 17.5	− 35.2	56.1	86.1	25.0	28.8
*Gynerium sagittatum*	− 43.5	− 47.8	− 48.1	− 58.8	− 32.0	− 34.2	− 34.8	− 42.0
*Philodendron corcovadense*	− 35.4	− 38.1	− 40.9	− 45.1	− 7.6	1.1	− 12.5	14.5
*Schoenoplectus californicus*	− 27.5	− 32.8	− 32.4	− 51.0	− 22.8	− 28.4	− 25.9	− 47.9
**Food species**								
*Acca sellowiana*	− 22.6	− 28.7	− 34.6	− 39.4	− 7.1	− 20.1	− 10.3	− 13.4
*Annona crassiflora*	− 68.2	− 82.9	− 76.3	− 91.8	− 61.0	− 77.9	− 71.5	− 89.1
*Araucaria angustifolia*	− 46.2	− 52.9	− 52.7	− 66.6	− 46.1	− 52.9	− 52.6	− 66.5
*Butia eriospatha*	− 67.7	− 77.3	− 67.8	− 87.4	− 45.2	− 50.4	− 52.7	− 64.3
*Campomanesia xanthocarpa*	− 38.9	− 32.1	− 36.6	− 47.8	− 35.5	− 25.8	− 35.2	− 43.3
*Eugenia involucrata*	− 42.4	− 44.5	− 45.6	− 61.8	− 39.9	− 40.4	− 43.6	− 59.7
*Eugenia pyriformis*	− 34.2	− 60.1	− 53.4	− 60.9	− 17.6	− 38.9	− 42.1	− 48.8
*Eugenia uniflora*	− 39.4	− 43.8	− 42.8	− 56.2	− 9.6	− 8.0	− 16.6	− 33.8
*Euterpe edulis*	− 50.1	− 52.7	− 58.0	− 69.7	− 19.9	− 19.9	− 20.3	− 33.7
*Opuntia elata*	− 12.0	− 58.6	− 57.8	− 47.9	45.8	− 28.7	− 26.2	12.3
*Passiflora actinia*	− 46.5	− 44.3	− 41.6	− 49.4	− 37.3	− 21.6	− 33.6	− 26.2
*Physalis pubescens*	− 43.2	− 48.1	− 47.1	− 61.9	− 41.4	− 46.0	− 45.5	− 59.2
*Plinia peruviana*	− 79.6	− 87.3	− 76.0	− 88.3	− 56.9	− 68.0	− 60.2	− 64.2
*Psidium cattleianum*	− 26.5	− 31.9	− 37.4	− 46.1	− 10.2	− 20.7	− 26.6	− 34.0
*Vasconcellea quercifolia*	− 42.1	− 51.5	− 52.8	− 50.3	− 26.3	− 43.0	− 44.5	− 34.8
**Forage species (Fabaceae)**								
*Adesmia bicolor*	− 64.5	− 69.0	− 74.1	− 80.1	− 48.2	− 58.5	− 48.6	− 76.0
*Adesmia latifolia*	− 68.3	− 47.2	− 51.8	− 52.9	40.8	39.3	13.8	23.5
*Adesmia tristis*	− 94.4	− 97.0	− 98.0	− 99.2	− 94.4	− 96.9	− 98.0	− 99.2
*Desmodium adscendens*	− 15.7	− 14.4	− 18.5	− 16.3	76.6	122.9	91.9	190.9
*Desmodium barbatum*	− 17.0	− 17.5	− 19.8	− 24.1	25.0	29.0	30.8	29.0
*Desmodium incanum*	− 27.7	− 29.9	− 31.9	− 41.9	− 21.5	− 24.5	− 27.6	− 37.7
*Desmodium subsericeum*	− 59.1	− 70.8	− 65.1	− 77.5	− 44.4	− 63.7	− 55.7	− 68.7
*Indigofera sabulicola*	0.3	0.3	0.5	0.0	183.7	218.4	191.0	387.7
*Leptospron adenanthum*	− 10.5	− 11.8	− 12.6	− 10.3	62.7	79.6	74.6	119.2
*Macroptilium psammodes*	− 20.9	− 30.3	− 32.3	− 12.9	115.3	123.1	115.5	227.9
*Ornithopus micranthus*	− 100.0	− 100.0	− 100.0	− 95.1	− 100.0	− 100.0	− 95.1	− 95.1
*Stylosanthes leiocarpa*	− 37.6	− 36.2	− 37.2	− 30.7	− 21.8	− 2.5	− 14.8	19.4
*Trifolium polymorphum*	− 47.1	− 48.8	− 67.6	− 81.8	2.6	2.3	− 5.0	− 48.1
*Trifolium riograndense*	− 91.1	− 75.5	− 95.3	− 86.4	− 91.1	− 75.2	− 95.3	− 86.4
*Vigna luteola*	− 16.9	− 23.1	− 21.7	− 26.2	0.5	− 4.7	− 5.8	− 11.8
**Forage species (Poaceae)**								
*Axonopus compressus*	− 36.4	− 34.8	− 37.5	− 55.2	− 23.8	− 19.7	− 22.6	− 42.0
*Axonopus fissifolius*	− 24.6	− 26.5	− 28.0	− 34.0	98.5	143.3	121.8	182.7
*Axonopus obtusifolius*	− 53.4	− 57.9	− 59.1	− 76.0	0.2	− 3.6	− 7.1	− 34.3
*Bothriochloa laguroides*	− 54.8	− 50.9	− 60.6	− 77.3	− 38.6	− 23.8	− 37.6	− 65.5
*Bromus auleticus*	− 81.0	− 82.3	− 69.7	− 88.5	− 79.2	− 80.3	− 52.3	− 83.6
*Bromus catharticus*	− 35.8	− 44.3	− 40.7	− 49.6	− 33.2	− 41.5	− 36.2	− 45.8
*Dichanthelium sabulorum*	− 35.4	− 38.2	− 45.1	− 62.9	− 34.4	− 33.9	− 44.0	− 60.3
*Echinochloa polystachya*	− 7.6	− 8.3	− 9.8	− 10.8	174.3	210.0	187.5	259.4
*Hemarthria altissima*	− 11.4	− 7.0	− 4.6	− 8.4	60.2	47.2	73.2	116.0
*Ischaemum minus*	− 29.9	− 40.9	− 47.1	− 6.0	42.2	36.8	22.6	141.5
*Mnesithea selloana*	− 41.1	− 49.9	− 64.4	− 72.4	36.3	28.6	30.0	23.3
*Nassella neesiana*	− 30.7	− 31.9	− 34.4	− 46.3	− 26.3	− 29.4	− 21.2	− 45.4
*Paspalum almum*	− 2.0	− 18.8	− 14.4	− 16.9	93.1	48.4	54.3	63.5
*Paspalum denticulatum*	− 22.9	− 31.1	− 31.2	− 43.5	− 1.8	0.6	7.8	− 17.9
*Paspalum dilatatum*	− 26.1	− 26.3	− 27.8	− 45.8	− 25.6	− 26.0	− 27.2	− 45.7
*Paspalum glaucescens*	− 59.0	− 42.0	− 55.2	− 61.0	− 36.7	5.7	− 37.8	− 41.9
*Paspalum guenoarum*	− 24.1	− 28.3	− 31.4	− 42.3	− 11.9	− 14.6	− 17.8	− 28.1
*Paspalum jesuiticum*	− 69.4	− 74.1	− 81.2	− 95.9	− 69.4	− 74.1	− 81.2	− 95.9
*Paspalum lepton*	− 5.6	− 6.6	− 12.3	− 5.4	117.6	111.5	137.9	127.1
*Paspalum modestum*	0.3	− 4.1	− 1.6	− 4.2	110.6	99.8	102.6	143.0
*Paspalum notatum*	− 26.0	− 27.1	− 28.0	− 42.0	− 25.2	− 26.0	− 26.9	− 40.6
*Paspalum pumilum*	− 41.8	− 39.3	− 39.7	− 50.2	− 41.0	− 34.7	− 38.5	− 47.9
*Paspalum regnellii*	− 16.8	− 37.4	− 29.0	− 40.4	40.4	17.0	7.5	20.1
*Poa lanigera*	− 30.2	− 28.6	− 30.3	− 46.7	− 3.2	− 9.1	4.1	− 25.3
*Schizachyrium tenerum*	− 49.8	− 51.2	− 53.4	− 68.9	− 46.3	− 46.2	− 50.0	− 64.3
**Medicinal species**								
*Achyrocline satureioides*	− 45.7	− 48.0	− 47.8	− 59.1	− 45.0	− 47.0	− 47.3	− 57.4
*Baccharis articulata*	− 48.8	− 53.2	− 50.9	− 58.7	− 44.2	− 43.7	− 45.1	− 53.3
*Baccharis crispa*	− 39.2	− 45.4	− 44.7	− 57.8	− 38.2	− 42.7	− 43.1	− 56.6
*Baccharis dracunculifolia*	− 42.9	− 52.8	− 51.6	− 64.5	− 37.4	− 44.7	− 45.6	− 58.4
*Bauhinia forficata*	− 42.5	− 44.9	− 43.4	− 55.3	− 33.0	− 32.4	− 33.1	− 45.1
*Bromelia antiacantha*	− 28.2	− 29.1	− 26.0	− 23.4	26.5	34.6	26.9	27.0
*Casearia sylvestris*	− 37.1	− 38.3	− 39.0	− 50.4	− 7.3	− 7.5	− 6.8	1.7
*Cecropia glaziovii*	− 42.6	− 51.8	− 55.4	− 69.1	− 21.7	− 41.0	− 40.3	− 54.2
*Copaifera trapezifolia*	− 19.3	− 22.3	− 25.1	− 39.6	20.1	9.5	15.0	− 17.5
*Croton celtidifolius*	− 35.0	− 54.2	− 44.0	− 81.6	− 32.8	− 53.3	− 40.6	− 81.4
*Cunila microcephala*	− 100.0	− 100.0	− 100.0	− 100.0	− 42.4	− 33.1	− 48.3	− 88.1
*Drimys brasiliensis*	− 73.8	− 76.9	− 77.9	− 91.7	− 73.8	− 76.9	− 77.9	− 91.7
*Echinodorus grandiflorus*	− 20.0	− 28.5	− 26.8	− 36.5	− 17.4	− 23.9	− 23.3	− 31.4
*Equisetum giganteum*	− 36.2	− 39.8	− 38.1	− 48.7	− 31.0	− 34.8	− 31.8	− 40.7
*Hypericum caprifoliatum*	− 5.5	− 11.2	− 12.8	− 19.9	29.6	7.2	2.6	5.1
*Ilex paraguariensis*	− 40.0	− 41.2	− 46.3	− 57.8	− 38.8	− 37.3	− 44.9	− 57.6
*Jodina rhombifolia*	− 57.5	− 58.4	− 59.5	− 62.2	− 51.5	− 52.5	− 53.0	− 50.9
*Mikania glomerata*	− 41.7	− 37.1	− 47.6	− 56.9	− 12.9	− 6.0	− 18.8	− 24.4
*Mikania laevigata*	− 27.4	− 23.0	− 26.5	− 32.8	− 12.8	3.2	− 14.4	− 4.5
*Monteverdia ilicifolia*	− 23.7	− 36.8	− 37.5	− 41.5	− 18.9	− 30.5	− 33.2	− 36.5
*Ocimum carnosum*	− 17.3	− 41.1	− 36.1	− 35.7	− 10.3	− 34.4	− 30.0	− 29.4
*Piper umbellatum*	− 43.7	− 50.4	− 48.9	− 63.7	− 37.6	− 42.2	− 45.0	− 54.4
*Plantago australis*	− 38.5	− 42.0	− 40.6	− 53.3	− 34.0	− 37.3	− 35.1	− 46.1
*Sambucus australis*	− 32.5	− 37.8	− 38.0	− 39.5	− 26.3	− 34.1	− 29.6	− 32.7
*Smilax campestris*	− 44.7	− 45.7	− 47.0	− 62.2	− 37.6	− 36.2	− 41.4	− 54.9
*Solanum mauritianum*	− 43.5	− 45.2	− 48.8	− 61.0	− 39.1	− 39.2	− 45.9	− 58.3
*Solanum paniculatum*	− 42.6	− 54.0	− 46.9	− 62.0	− 26.4	− 38.2	− 30.5	− 30.1
*Sorocea bonplandii*	− 43.8	− 56.4	− 52.3	− 68.1	− 21.1	− 39.5	− 21.9	− 58.3
*Trichilia catigua*	− 38.5	− 66.0	− 56.5	− 79.4	− 8.5	− 34.9	− 23.5	− 51.8
*Varronia curassavica*	− 29.1	− 33.2	− 32.5	− 36.5	65.3	122.4	83.6	229.5
*Wilbrandia ebracteata*	− 36.3	− 28.6	− 23.2	− 60.5	28.2	20.5	42.3	− 33.6
*Zollernia ilicifolia*	− 38.8	− 42.5	− 42.4	− 57.4	− 14.1	− 6.6	− 15.0	− 36.1
**Ornamental species**								
*Ananas bracteatus*	− 44.6	− 46.6	− 46.0	− 60.2	− 37.3	− 37.7	− 39.5	− 52.7
*Aspilia montevidensis*	− 27.5	− 28.9	− 35.6	− 27.6	− 2.3	9.9	− 10.8	13.4
*Calliandra tweedii*	− 32.7	− 42.9	− 42.4	− 52.5	− 27.8	− 33.9	− 38.1	− 44.5
*Cortaderia selloana*	− 35.0	− 44.0	− 39.2	− 57.7	− 30.1	− 39.4	− 31.6	− 52.6
*Dyckia distachya*	− 44.7	− 99.6	− 71.8	− 91.7	159.0	− 36.8	44.4	21.4
*Epidendrum fulgens*	− 18.1	− 19.0	− 37.5	− 3.7	30.3	123.1	14.2	264.0
*Fuchsia regia*	− 51.2	− 56.4	− 56.9	− 79.9	− 49.7	− 54.9	− 55.6	− 79.3
*Gomesa flexuosa*	− 38.1	− 40.6	− 39.2	− 60.5	− 23.0	− 31.4	− 24.6	− 52.4
*Handroanthus chrysotrichus*	− 38.5	− 43.2	− 45.3	− 60.5	− 14.9	− 25.0	− 21.0	− 40.7
*Heliconia farinosa*	− 13.7	− 15.5	− 12.6	− 20.6	27.7	64.1	71.9	29.1
*Jacaranda puberula*	− 40.2	− 49.4	− 50.1	− 63.7	− 27.0	− 36.9	− 36.2	− 52.5
*Parodia ottonis*	− 63.9	− 56.0	− 72.6	− 82.5	− 51.8	− 42.4	− 68.5	− 76.6
*Petunia integrifolia*	− 4.2	− 5.6	− 12.7	− 12.2	7.3	1.2	− 10.1	− 4.9
*Pyrostegia venusta*	− 49.3	− 46.6	− 46.3	− 48.9	− 12.0	22.2	8.4	70.5
*Rumohra adiantiformis*	− 34.9	− 38.3	− 41.6	− 52.2	− 34.6	− 36.6	− 41.5	− 51.8
*Syagrus romanzoffiana*	− 61.0	− 62.4	− 61.8	− 75.5	− 27.3	− 30.0	− 21.7	− 56.0
*Tibouchina sellowiana*	− 41.8	− 53.9	− 50.3	− 74.5	− 9.1	− 39.6	− 35.0	− 69.8
*Trichocline catharinensis*	− 54.1	− 85.6	− 83.3	− 89.0	− 53.0	− 71.2	− 83.3	− 86.7
*Verbena rigida*	− 47.5	− 68.4	− 67.0	− 69.3	− 32.6	− 50.8	− 49.8	− 66.2
**Timber species**								
*Apuleia leiocarpa*	− 56.0	− 61.0	− 60.6	− 75.3	− 52.8	− 57.0	− 57.8	− 71.1
*Aspidosperma polyneuron*	− 49.7	− 57.1	− 47.7	− 63.6	34.7	41.4	29.4	155.9
*Ateleia glazioveana*	− 28.3	− 15.3	− 36.7	− 17.0	17.3	39.2	− 11.5	27.4
*Balfourodendron riedelianum*	− 29.0	− 52.7	− 37.7	− 51.6	− 24.2	− 47.3	− 31.1	− 46.0
*Cabralea canjerana*	− 42.4	− 52.0	− 50.9	− 61.6	− 40.4	− 48.1	− 48.5	− 57.1
*Calophyllum brasiliense*	− 39.8	− 41.2	− 43.3	− 40.7	104.5	137.5	125.3	183.0
*Cedrela fissilis*	− 48.4	− 54.1	− 52.8	− 67.1	− 47.8	− 52.6	− 52.5	− 66.3
*Colubrina glandulosa*	− 60.7	− 59.6	− 54.9	− 74.7	− 52.3	− 49.9	− 45.3	− 69.8
*Cordia trichotoma*	− 54.0	− 59.9	− 58.4	− 72.6	− 41.5	− 45.2	− 44.8	− 57.6
*Enterolobium contortisiliquum*	− 46.8	− 63.3	− 55.7	− 74.8	− 23.9	− 44.9	− 32.0	− 49.3
*Handroanthus heptaphyllus*	− 26.4	− 30.7	− 38.3	− 58.2	28.0	25.7	7.3	− 12.4
*Hyeronima alchorneoides*	− 34.8	− 38.2	− 36.3	− 45.4	− 1.1	13.7	9.7	32.6
*Miconia cinnamomifolia*	− 8.9	− 15.5	− 14.6	− 27.0	27.9	12.4	15.0	− 13.0
*Mimosa scabrella*	− 44.8	− 61.5	− 61.2	− 83.5	− 44.8	− 61.4	− 61.2	− 83.5
*Nectandra lanceolata*	− 40.2	− 52.1	− 49.7	− 60.8	− 34.6	− 47.3	− 44.9	− 56.9
*Ocotea puberula*	− 38.2	− 39.7	− 39.7	− 55.4	− 34.0	− 33.3	− 35.4	− 50.8
*Parapiptadenia rigida*	− 38.4	− 48.3	− 44.5	− 56.5	− 20.9	− 17.1	− 26.8	− 33.5
*Peltophorum dubium*	− 76.5	− 79.4	− 74.8	− 88.4	− 58.3	− 61.5	− 49.8	− 68.9
*Piptocarpha angustifolia*	− 41.7	− 65.7	− 69.9	− 72.2	− 9.2	− 47.3	− 59.5	− 56.3
*Schizolobium parahyba*	− 38.6	− 36.0	− 36.7	− 52.2	− 24.1	− 11.1	− 15.9	− 39.1

**Figure 2:**
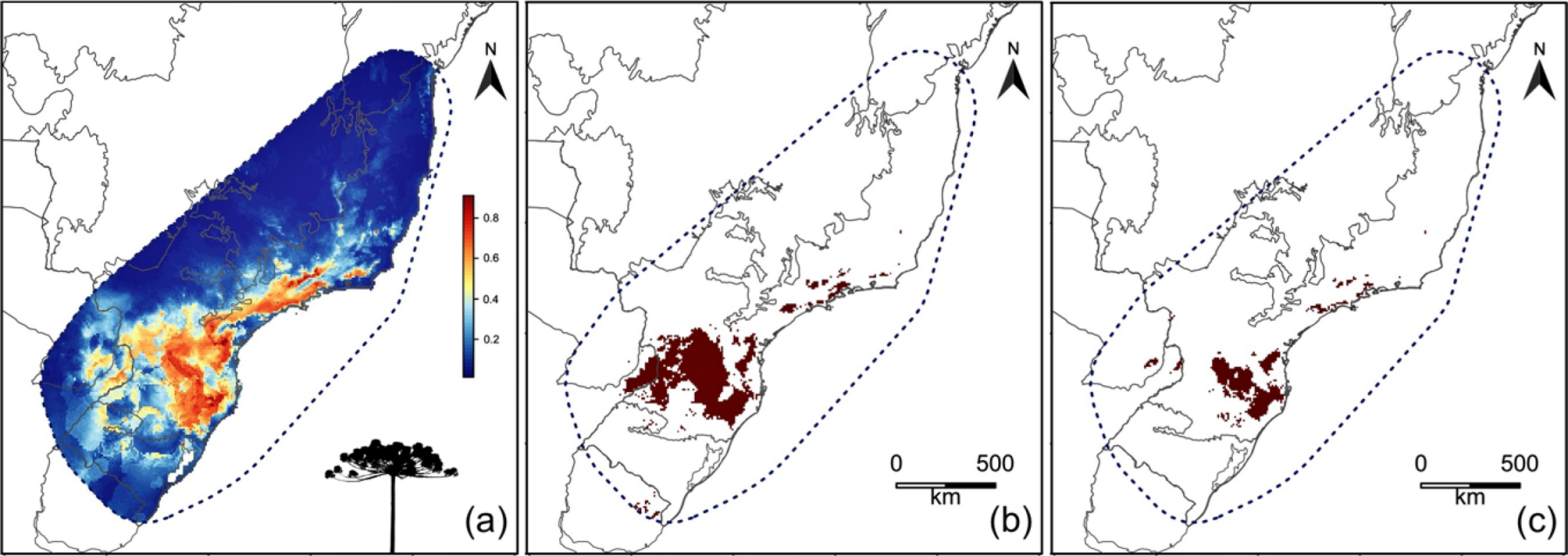
Decline of BAH by climate change for *A. angustifolia*, a species traditionally combined with other agricultural crops from Atlantic Forest. **a)** Current habitat suitability. Blue to red indicates the increase of suitability **b-c)** Future suitable habitats based on SSP2-4.5 and SSP5-8.5 scenarios for 2061–2080. Wine red colour indicates the remaining BAH. Estimated BAH is surrounded by dotted lines in royal blue colour. Maps created with custom R script. Version R 4.1.1 (https://www.R-project.org/). Base map source (Terrestrial biomes shapefile) obtained from the Brazilian Ministry of the Environment (https://www.gov.br/mma/).

Looking at specific groups by their main use, we estimate loss of BAH ranging from 2,9% (*Tropaeolum pentaphyllum)* to 76.5% (*Pimenta pseudocaryophyllus*) for aromatic species; 6.1% (*Geonoma gamiova)* to 58.8% (*Gynerium sagittatum)* for fibrous species; 7.1% (*Acca sellowiana)* to 91.8% (*Annona crassiflora)* for food species; 2.5% (*Stylosanthes leiocarpa)* to 100% (*O. micranthus)* for forage species from Fabaceae family; 1.6% (*Paspalum modestum*) to 95.9% (*Paspalum jesuiticum*) for forage species from Poaceae family; 4.5% (*Mikania laevigata*) to 100% (*C. microcephala*) for medicinal species; 2,3% (*Aspilia montevidensis*) to 99.6% (*Dyckia distachya*) for ornamental species; and 1.1% (*Hyeronima alchorneoides*) 88,4% (*Peltophorum dubium*) for medicinal species (Table 1, Supplementary Table S1). The species *Araucaria angustifolia*, which has already been traditionally combined in agroecological practices in southern Brazil is predicted to reduce up to 66% of its BAH under SSP5-8.5 by 2061–2080 in both dispersal scenarios (Fig. 2, Supplementary Table S1).

### IUCN Red list preliminary assessment

**Figure 3:**
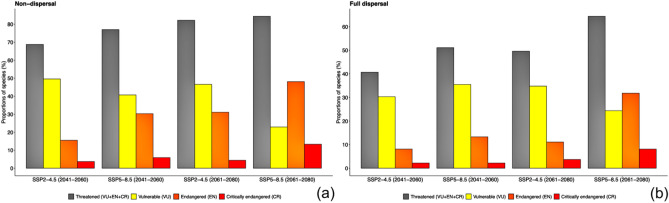
Percentage of Brazilian agroforestry plant species potentially qualified in a threat categories VU + EN + CR, and in separate categories Vulnerable (VU), Endangered (EN) and Critically Endangered (CR), under two different climate change scenarios (SSP2-4.5 and SSP5-8.5) for the following time periods: 2041–2060 and 2061–2080, assuming two dispersal scenarios: (**a**) non-dispersal and (**b**) full dispersal.

**Figure 4:**
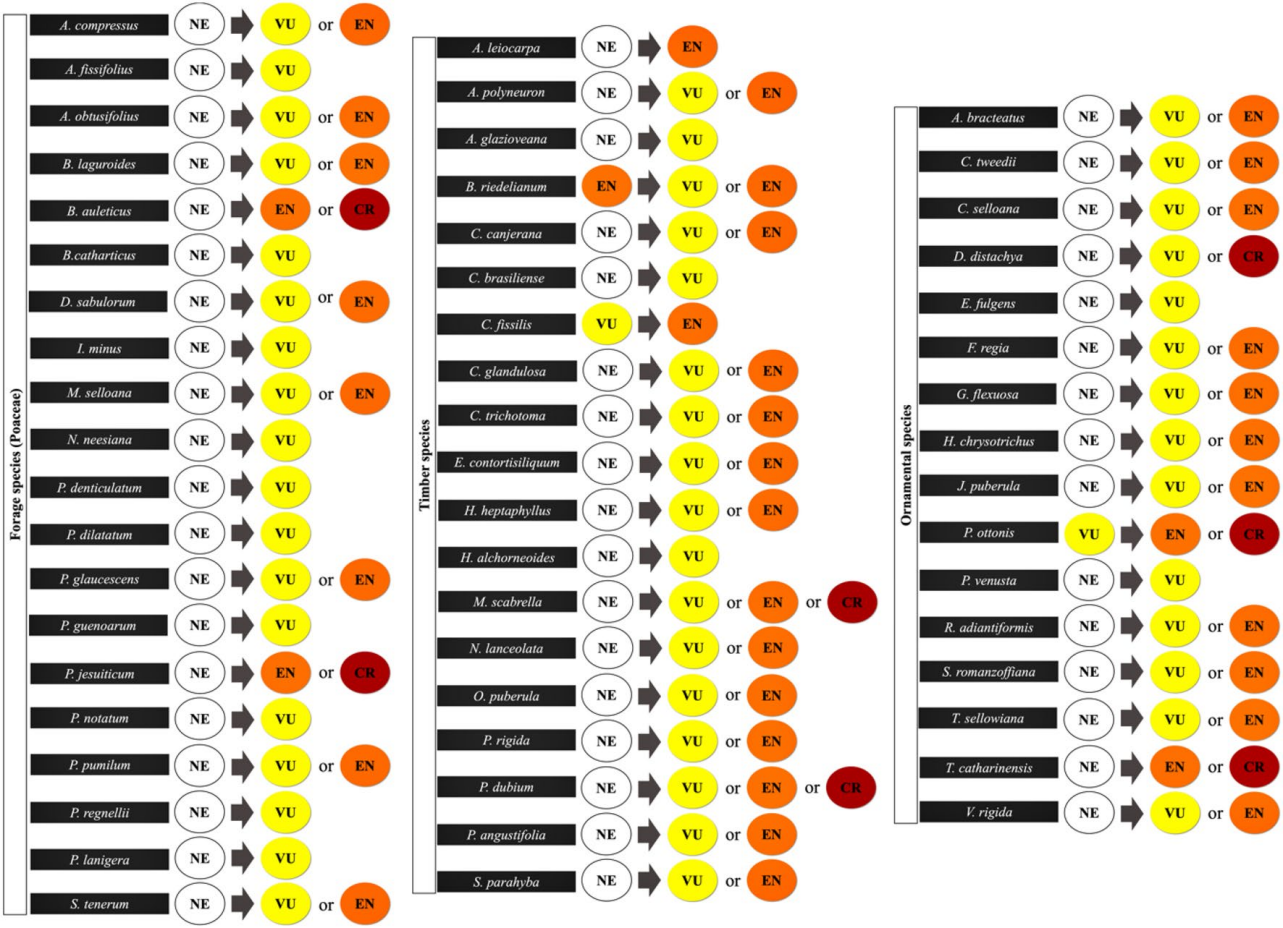
Native potential agroforestry plant species changing from a current assessed IUCN category or Not Evaluated (NE) to a threat category, Vulnerable (VU), Endangered (EN) and Critically Endangered (CR), based on different major uses due to climate change.

Assessing the geographic range (B1a + B2a criteria), we found that only 4.3% of the native species was qualified for a Threat category (Supplementary Table S2). However, when considering the predicted quantitative habitat loss due to climate change (A3c criterion) the percentages increased. We observed that 68.8% (SSP2-4.5/2041–2060) to 84.4% (SSP5-8.5/2061-2080) under the non-dispersal scenario (Fig. 3a, Supplementary Table S1) and 40.7% (SSP2-4.5/2041-2060) to 64.4% (SSP5-8.5/2061-2080) under the full dispersal scenario of the species could be qualified as threatened according to IUCN Red List criteria (Fig. 3b, Supplementary Table S1). The highest proportions of species were qualified as vulnerable with 49.6% (SSP2-4.5/2041-2060), 40.7% (SSP5-8.5/2041-2060) and 46.6% (SSP2-4.5/2061-2080) under the non-dispersal scenario and 30.3% (SSP2-4.5/2041-2060), 35.5% (SSP5-8.5/2041-2060) and 34.8% (SSP2-4.5/2061-2080) under the full dispersal scenario. The exception was the SSP5-8.5 (2061-2080) scenario, where the largest proportion was qualified as endangered in both the non-dispersal scenario (48.1%) and full dispersal scenario (31.8%) (Fig. 3a,b). Approximately 96% of the species have not yet been accessed by the IUCN and are currently Not Evaluated (NE). Here, we show that 86% of these species are predicted to change from Not Evaluated to a threat category, Vulnerable (VU), Endangered (EN) and Critically Endangered (CR), in all major uses and based on different climate change scenarios (Fig. 4, Table 2, Supplementary Fig. S1). All species groups changed from 50 to 100% of their species to the Vulnerable category. Timber species had the highest percentage of their species (84.2%) changing to the Endangered category and, the forage species belonging to the Fabaceae family had the highest (55.6%) changing to the Critically Endangered category.

**Table 2:** Number and percentage of species changing from Not Evaluated to a threat category, Vulnerable (VU), Endangered (EN) and Critically Endangered (CR), in all major uses and in at least one climate change scenario.

Main use	VU		EN		CR	
	*n*	%	*n*	%	*n*	%
Aromatic species	4	100.0	2	50.0	–	–
Fibrous species	4	100.0	2	50.0	–	–
Food species	12	80.0	11	73.3	3	20.0
Forage species (Fabaceae)	6	66.7	5	55.6	5	55.6
Forage species (Poaceae)	10	50.0	9	45.0	2	10.0
Medicinal species	27	93.1	20	69.0	3	10.3
Ornamental species	14	87.5	12	75.0	3	18.8
Timber species	17	89.5	16	84.2	2	10.5

## Discussion

Climate change is an important driver of species extinction^4,25^. We found that future climate change was predicted to cause a decline in BAH between 38.5–56.3% under the non-dispersal scenario and between 22.3–41.9% under the full dispersal scenario in 135 Brazilian native species. Several studies forecasted the impacts of climate change on species distribution by using ENMs worldwide^44–47^, and consequently on ecosystem functionality^4,48^. The worst-case scenario (SSP5-8.5) showed the highest average decline in BAH in both non-dispersal (56.3%) and full dispersal (41.9%), when compared to the stabilization scenario (SSP2-4.5) and in 2061–2080, showing that species tend to be more threatened in this scenario and year, as demonstrated in other studies conducted with Brazilian species^49,50^. Our study evaluated widespread plant species, such as *Axonopus fissifolius*, occurring in all Brazilian phytogeographic domains and species with a narrow-ranged distribution, such as *Adesmia bicolor* from South Brazilian grasslands (Pampa). We noticed that both narrow-ranged and widespread species may be impacted by climate change. For instance, the narrow-ranged species *O. micranthus*, used in annual forage crops, is predicted to lose up to 100% of its suitable habitat in most climate change scenarios. Our models also suggested that the widespread Brazilian peppertree (*Schinus terebinthifolia*) may lose up to 47% of its original habitats. These results need to be interpreted with caution, since all species that were predicted to lose 100% of BAH have small sample sizes (n < 50, Supplementary Table S1), which is one of the main determinants of model accuracy^51^. Some species tend to be favored by certain climate change scenarios under full dispersal scenario^52,53^, such as here the medicinal species *Varronia curassavica*, with an increase of BAH up to 230%; the ornamental species *Epidendrum fulgens* up to 264% and the forage species *Indigofera sabulicola* up to 387%. Although some species were predicted to expand their range, this does not guarantee the survival of these species, since other drivers, such as the capability of poor dispersal species to cope with climate change^54^, deforestation^44,55^ and other land-use changes threaten Brazilian ecosystems and the survival of their associated species^56^.

Rapid range changes of species may, in turn, impact material, non-material and regulating contributions of nature to people^2,48,57,58^. The global productivity of farms may be negatively affected by climate change throughout most of the tropics^59^. Smallholders and traditional Brazilian communities such as the *caiçaras*, *quilombolas* and indigenous peoples make use of these species in agroforestry practices^13^, a land use management system that increases carbon sequestration in biomass and soils^24,60^. These systems improve people’s livelihoods by simultaneously providing income, food security, fuel, medicine, forage, and/or other goods and services. Moreover, increased tree cover due to agroforestry may help to mitigate climate change^23,57,61^. Here we present evidence of how currently suitable areas for cultivation of these species may become unsuitable in the future. This may lead to a severe decline in people's livelihoods and regional food security^23,62^. Moreover, under climate shifts species survival is likely to be threatened by biogeographic barriers such as an agricultural or otherwise ecologically degraded landscape matrix which would prevent species migration to climatically more suitable areas^63^.

The outcomes of climate change predicted by biogeographical and ecological studies have been neglected and have barely been integrated into conservation planning^30,64^. Prioritization of conservation efforts is often based on a species’ extinction risk^65^. Determining whether a taxon is threatened with extinction depends on biological indicators, such as rapid population decline, and qualifying species in a threat category may assist in decision making^43^. Our analyses highlight that 68.8% to 84.4% (non-dispersal) and 40.7% to 64.4% (full dispersal) of our species of interest may become threatened. Additionally, species already threatened according to the IUCN red list; *A. angustifolia* (CR), *Butia eriospatha* (VU), *Balfourodendron riedelianum* (EN), *Cedrela fissilis* (VU) and *Parodia ottonis* (VU) will remain listed as such as a result of climate change. In assessing Amazonian tree species, Gomes et al. (2019) found that 43–46% of trees species should be listed as threatened according to IUCN A2, A4, B1 and D2 criteria for RCP 2.6 and RCP 8.5 respectively. Similarly, Zizka et al*.* (2020), analyzing the conservation status of species of Bromeliaceae based on the geographic range in the Americas, showed that a total of 81% of bromeliad species are possibly threatened according to IUCN red list criteria. These authors observed that the medicinal species *Bromelia antiacantha* is possibly not threatened (LC or NT) in the current scenario, which agrees with our full dispersal scenario results that show that the BAH of this species may remain stable under all future climate change scenarios as well. Elias et al.^66^ assessed the conservation status of eleven palm species through A2, A4 and B2 criteria in the state of Santa Catarina (Atlantic Forest) and qualified *Euterpe edulis* and *G. gamiova* as Vulnerable and *Butia catarinensis* and *Butia eriospatha* as Endangered. According to our findings, *E. edulis* may be categorized as Endangered for showing a decline in BAH of over 50% in all climate change scenarios when assuming species have no dispersal. However, when we considered the full dispersal scenario, we noted that although there are declines in BAH in all climate change scenarios, only in the SSP5-8.5 for 2061–2080 this species might be qualified as Vulnerable. For *G. gamiova* we observed a similar result (Vulnerable) exclusively in the SSP5-8.5 for 2061–2080 assuming no dispersal. On the other hand, assuming the full dispersal scenario, we noted an increase in BAH of up to 86% in the SSP5-8.5 for 2041–2060 for all climate change scenarios. Our results are equally consistent with the assessment of these authors for *B. catarinensis* and *B. eriospatha*. We still recorded an alarming scenario for *B. eriospatha,* qualifying the species as Critically endangered because of a decline of BAH declines over 87% (non-dispersal) under the worst-case scenario SSP5-8.5 for 2061–2080. Thus, given the large number of agroforestry species at risk of extinction, and the low number of species assessed by the IUCN Red List, we emphasize an urgent need for updates of the official list of threatened species, to provide a more precise indicator for threatened plant species conservation planning in Brazil. Despite all legal implications for threatened species in Brazil, agroforestry systems can act as an alternative to overcome part of the conflicts between conventional agricultural production and the conservation of natural resources, as can be seen in the new forest code (Law 12.651/2012), which provides explicit provisions for sustainable agroforestry. Additionally, a specific Law (12.854/2013) promotes forest recovery activities and the implementation of agroforestry systems^34^.

It is well known that deforestation is a primary driver leading species to extinction^67^. However, climate change is expected to overtake this driver in a few decades^44^. The majority of the species prioritized in the “Plants for the future initiative”, found mainly in the Atlantic Forest have great potential to be conserved through sustainable practices, particularly by smallholders and traditional communities^13^. The Atlantic Forest remains endangered as a result of continued deforestation and the future of this forest relies on well-structured conservation plans based on reliable information^55,68,69^. The Atlantic Forest cover has been reduced to less than 20% of its original size^70,71^, distributed mainly in small and disturbed fragments of less than 50 hectares^56,72^. Thus, most of the endemic species in the Atlantic Forest biome could already be qualified as critically endangered according to IUCN criteria^43^. On the other hand, despite our predicted catastrophic scenarios for native Atlantic Forest species, we observe that species such as Bracatinga (*M. scabrella*), Brazilian peppertree (*Schinus terebinthifolia*) and Peruvian groundcherry (*Physalis peruviana*), are distributed across the neotropics and easily found along streets and city squares all over Brazil. Many of these are pioneers, some almost ruderal, regenerating easily in open city areas^13,29^. Many of these forest species have endured over 500 years of deforestation, and still remain abundant in the Atlantic Forest even after losing approximately 80% of their natural habitat^29,56,71^. Hence, we note the need for studies that address species response to global changes to better understand the resilience potential of these species.

Protecting people’s livelihoods in a rapidly changing climate may be one of the great challenges of the twenty-first century. Although it is not shared by all of the scientific community, as discussed by Loreau^73^, species with economic value seem to have advantages for conservation over those with non-economic value as can be seen in long-term human activities such as protection, transport and planting of useful species and removal of non-useful species by local communities^31,42,74^. Indeed, socioeconomic underutilization of plant resources may in some cases even jeopardize socioecological synergies of tropical forest resilience^58^. All species analyzed here have a great potential to be conserved through a conservation-by-use approach, because of their different uses that do not necessarily jeopardize reproduction and persistence. They play an important role among local communities^41,48,50,75,76^ and farmers’ livelihoods^31,42^. Searching for evidence of conservation among species with economic and cultural values, Reis et al.^42^ noticed that the species *Ilex paraguariensis*, *A. angustifolia* and *B. antiacantha* were intentionally favored through protection, transplantation and selection by farmers. Furthermore, Donazzolo et al.^32^ noted that management of *Acca sellowiana* populations, retained high level of genetic diversity and tended to increase the species genetic variability. We argue that adopting measures, such as the establishment of new agroforestry systems to increase carbon sequestration, the selection of varieties capable of withstanding new climates and the improvement of habitat connectivity to facilitate species migration/dispersal should be a strategy for short-term and long-term conservation and people’s livelihoods.

ENMs are widely used to forecast the distribution of species across geographic space and time. Building meaningful models to estimate the future distribution of species for an uncertain future requires very specific decisions and interpretations with extreme caution^51,77,78^. Several uncertainties and complexities are related to our study. Modeling a large number of species can make the species-specific selection of predictors methodologically and practically complex^77,79^. To mitigate this, we selected the most suitable environmental predictors for different plant growth forms. Model performance evaluation is a key step for ENM studies and probably the most problematic one owing to its complexity^79^. The random cross-validation approach is the most common practice, adopted by modelers to evaluate model performance, where datasets are split into k folds, using one part to test the model and the remaining (k-1 folds) to calibrate the model^80,81^. To reduce the over-optimistic nature of cross-validation, we applied a null-model for significance testing of presence-only ENMs^82^. Binarization of continuous probabilities output is commonly employed by modelers to quantify species range changes and build species richness over time^83^. Nevertheless, Santini et al.^51^ recently concluded that this practice reduces the predictive probability of models. Although we binarized ENMs outputs to quantify the climate change impacts, we applied a threshold highly indicated for conservation purposes for showing high performance in the identification of suitable areas and commonly used^50,84–86^. We assumed that the species are at equilibrium with the environment^87^ and occurrence records were sampled randomly^88^. Furthermore, we included no biotic interactions^40,89^, adaptations and evolution^90^ into our modeling approach. For dispersal^37,40^, we considered two scenarios (non-dispersal and full dispersal), but we limited the full dispersal scenario to species BAH, since we understand that plant dispersal rates over 0.1 km/year might not occur for vascular and non-vascular plants^5,64,91^ or over 100 km for Brazilian tree species as result of climate change^44,92^. Although humans fundamentally affect dispersal and alter landscapes by transporting individuals^93–95^, we did not include human-mediated dispersal data in our models due to the lack of information related to human migrations as well as for each specific species. Another limitation of our study is to restrict our analysis to the estimated BAH of species, which may mask some macroecological patterns, yet adopting this conservative approach allows us to observe more concise species responses and diminish model overfitting^36,44,96^.

In summary, we showed that future climate will likely trigger a decline in BAH between 38.5–56.3% under the non-dispersal scenario and between 22.3–41.9% under the full dispersal scenario of several native potential agroforestry species from the Brazilian flora. Additionally, we found that only 4.3% of the studied species could be threatened under the IUCN criteria B1 and B2. However, when considering the IUCN criterion A3, 68.8–84.4% (non-dispersal) and 40.7–64.4% (full dispersal) of our species of interest could be qualified as threatened. Although accessing genetic material with quality for native species might be difficult and the scenarios used here estimate considerable losses for 2041–2060 and 2061–2080, we argue that actions such as the promotion of these species in agroecosystems are promising alternatives to increase their population sizes. We urge that public policies involving farmers and local communities be adopted, as practices and management systems implemented by them have proven to maintain landscapes with productive forest fragments, and consequently favors species and forest conservation. Lastly, we highly recommend the development of scientific research towards biotechnological applications to select promising genotypes for a changing global climate.

## Methods

### Study area and target species

The study area includes the Atlantic Forest and Pampa grasslands in Eastern South America. We modelled 139 native potential agroforestry plant species (i.e. aromatic, fibre, food, forage, medicinal, ornamental and timber species) prioritized by the Brazilian Ministry of the Environment initiative “*Native species of the Brazilian flora of current and potential economic value—Plants for the Future—Southern Region*” (*Espécies Nativas da Flora Brasileira de Valor Econômico Atual e Potencial – Plantas para o futuro – Região Sul*). This initiative seeks to promote the sustainable use of Brazilian native plant species often used in different regions of the country^13^. In addition to contributing to the country's commitments under the Convention on Biological Diversity (CBD) and International Treaty on Plant Genetic Resources for Food and Agriculture, particularly with regard to promoting the sustainable use of biodiversity components^13^, these species provide food security for local communities and have commercial value in national and foreign markets. In spite of the fact that not all species have already been found in current agricultural systems or been managed by farmers, they all have one or multiple uses and can be combined in mixed cropping. Some species such as *A. angustifolia*^31^, *A. sellowiana*^32^, *E. edulis*^33^, *Ilex paraguariensis*^34^, and *Mimosa scabrella*^35^ have already traditionally been combined with other agricultural crops in managed landscapes in southern Brazil. All taxonomic authorities and species common names are listed in Supplementary Table S3.

### Species occurrence data

Occurrence data for the 139 species evaluated here was downloaded from the Global Biodiversity Information Facility (https://doi.org/10.15468/dl.vjezvb) ^97^. We collected a total of 28,860 unique records. The sample size for species ranged from 12 (*Ornithopus micrantus*) to 5464 (*Casearia sylvestris*). We standardized botanical names using the R package ‘flora’^98^, which uses the nomenclature accepted by the Brazilian Flora 2020 project (http://floradobrasil.jbrj.gov.br/). To avoid modeling truncated niches, we extracted all records from an extent, defined by latitudes 60°S-15°N and longitudes 90°-30°W^99^. We checked the geographical consistencies of all records using the cleaning pipeline proposed by Gomes et al.^36^. Firstly, we removed all occurrences outside the Neotropics. Then, we removed all records with missing latitude and longitude, using the function ‘cleancoordinates’ from the R package ‘CoordinateCleaner’^100^. Finally, we estimated the kernel density for each species in order to remove spatial outliers using the density function from the R package ‘stats’^101^. As geographic sampling biases are common among biological collections^102,103^, which can lead to over-representation of environmental conditions, we spatially filtered the species occurrence data over a distance of 20 km using the R package ‘spThin’ in order to diminish spatial autocorrelation^104^. We did not model species for which there were less than ten records, as models fit with few data may not be reliable^105,106^.

### Environmental predictors

We obtained 19 bioclimatic variables from the Worldclim version 2.1. (http://worldclim.org) at a resolution of 5 arc-minutes (roughly 10 km at the equator), to characterize the species climatic requirements^107^. These environmental variables represent the time period of 1970–2000. Predictors were selected (1) a priori based on their biological significance for different plant growth forms^108^ (Supplementary Table S4). These predictors are critical in determining the distribution limits of a wide range of plant growth forms and are highly related to plant physiological responses^109,110^. We then (2) checked for multicollinearity by examining the correlation structure of the predictor variables through the variance inflation factor (VIF) for epiphyte, fern, graminoid, herb, hydrophyte, lithophyte, shrub, tree and vine species^111^. This measure evaluates how much the variance of an estimated regression coefficient increases if their predictors are correlated^112^. We kept only predictors with VIF values below 5^113^. The VIFs were checked using the function ‘vifstep’ in the R package ‘usdm’^114^. The retained predictors are shown in Supplementary Table S5.

### Modeling approach

We used bioclimatic habitat suitability to assess the potential impacts of climate change on species’ BAH and inform IUCN Red List assessments^115^. To delimit BAH, we incorporated a 100 km buffer around species extent of occurrence (EOO), as we understand that plant dispersal rates over 0.1 km/year might not occur for vascular and non-vascular plants^5,64,91^, and adopting a conservative approach reduces model overfitting^36,44,96^. EOOs were quantified by drawing a minimum convex polygon (MCP) around known species records as recommend by IUCN^43^. Current and future potential habitats for species were predicted using MaxEnt v.3.4.1 k, a machine-learning algorithm used to estimate species' probability distribution^116^. MaxEnt is the presence-based method widely used for having high performance when compared to other available algorithms^36,105,117–119^. ENMs were fitted using the following parameters in the MaxEnt: bootstrap method with 100 replicates, 500 maximum iterations, 10,000 points of background, and Cloglog output format. We only kept linear and quadratic features to avoid overfitting of the models and as recommended by Merow et al*.* because of the absence of a biological justification with the variables used^120,121^. Furthermore, we inspected species-response-curves to avoid spurious calibrations, following the evaluation strip method proposed by Elith et al.^122^. This method investigates the effect of one variable at a time, keeping the others constant at their mean values^122^. To assess robustness and alert policy-makers for the uncertainties typically associated with these methods, each ENM was tested against a bias corrected null-model as proposed by Raes and ter Steege^82^. The AUC values of the ENMs built with *n* occurrence records were tested against the upper AUC values of the lower quantile of 95% of the AUC values obtained from 100 × *n* points drawn and predicted randomly. Only significant ENMs were projected to future climatic conditions.

### Future projections

The climate projections were carried out according to the Sixth Assessment Report (AR6) of the IPCC, using two Shared Socioeconomic Pathways (SSPs) as reference (SSP2-4.5 and SSP5-8.5). SSPs are projections of future climates, based on different socioeconomic assumptions such as population, technological, and economic growth. The SSP2-4.5 (2041–2060) is a stabilization scenario, assuming global temperature increases ranging from 2.1 to 4.3 °C and mean warming of 3.0 °C. The SSP5-8.5 (2061–2080) represents the worst-case scenario, assuming absence of climate change policies, with global temperatures continue to rise throughout the twenty-first century, with estimates ranging from 3.8 to 7.4 °C and mean warming of 5.0 °C^9^. We averaged eight different global climate models: BCC-CSM2-MR, CNRM-CM6-1, CNRM-ESM2-1, CanESM5, IPSL-CM6A-LR, MIROC-ES2L, MIROC6 and MRI-ESM2-0 to take into account the uncertainties related to future climate conditions^81^. The fitted consensus ENMs were projected to these two datasets to obtain predicted future maps of habitat suitability for each species.

To map changes in future ranges of species, we converted the continuous habitat suitability into binaries using the maximum training sensitivity plus specificity threshold^85,123^. This threshold is indicated for conservation purposes for its high performance in the identification of suitable areas^50,84–86^. To assess whether species will face a decline or expansion in BAH under future climate conditions, we quantified the difference between the relative number of pixels occupied in current and future BAHs. We assumed that species have no dispersal capacity and full dispersal capacity for 2041–2060 and 2061–2080 timeframes. In the first scenario, species do not have the ability to disperse and reach new areas (pixels) in future climate scenarios. In the second, species can migrate within the estimated BAH of each species. Here, to ensure transparency and reproducibility for reporting ENMs, we adhered to the ODMAP (Overview, Data, Model, Assessment, Prediction) protocol v1.0. (Supplementary Table S6), as proposed by Zurell et al.^78^. All analyses were conducted within R environment version R 4.1.1^101^.

### IUCN Red list preliminary assessment

The IUCN Red List assessments provide important information related to species status, trends and threats for the establishment of conservation planning and improvement of decision-making^43,124,125^. Criterion B is linked to the geographic range and has two sub-criteria (B1 and B2), which are based on the extent of occurrence (EOO) and B2 on the area of occupancy (AOO), respectively^43^. Further, three other conditions (a, b, and c) describe aspects of the biology and potential decline of the taxon in response to threats^43^. At least one sub-criterion and two conditions must be met to qualify a given species as threatened^43^. Following the guidelines for using the IUCN Red List Categories and Criteria version 14, we calculated the geographic range (B1a + B2a criteria) using the R package ‘ConR’^126^. Additionally, we evaluated predicted quantitative habitat loss due to climate change by assessing the decline in habitat quality (A3c criterion), suspected to be met in the future, to qualify whether a particular species would be in a threat category^43^. The classification of threat includes: Vulnerable (VU), Endangered (EN) and Critically Endangered (CR). To qualify a species as Vulnerable, qualitative habitat loss must be ≥ 30%, Endangered ≥ 50% and Critically Endangered ≥ 80%. These categories are related to the risk of extinction of species in the wild^43^.

## Supplementary Information

Supplementary Information.

## Data Availability

Datasets generated and codes are available on GBIF https://doi.org/10.15468/dl.vjezvb and on GitHub repository https://github.com/vplima/ENM.git.
