# Supplementary material for: Climate change threatens native potential agroforestry plant species in Brazil
Source: Sci Rep. 2022 Feb 10;12:2267. doi: 10.1038/s41598-022-06234-3 (PMC8831634; doi:10.1038/s41598-022-06234-3)
Supplement: Supplementary file 1 — Supplementary Information. [file 41598_2022_6234_MOESM1_ESM.docx]

**Online supplementary information for “Climate change threatens native potential agroforestry plant species in Brazil”**

Table S1. Number of unique records, significant models, species future range changes and species qualified for the IUCN threatened category (VU, EN, CR) based on A3 criterion under two dispersal scenarios.

|  |  |  | **NON-DISPERSAL** | | | | | | | | **FULL DISPERSAL** | | | | | | | |
| --- | --- | --- | --- | --- | --- | --- | --- | --- | --- | --- | --- | --- | --- | --- | --- | --- | --- | --- |
|  |  |  | 2041-2060 | | | | 2061-2080 | | | | 2041-2060 | | | | 2061-2080 | | | |
|  | Records | Significant ENM | SSP2-4.5 | | SSP5-8.5 | | SSP2-4.5 | | SSP5-8.5 | | SSP2-4.5 | | SSP5-8.5 | | SSP2-4.5 | | SSP5-8.5 | |
| ***Aromatic species*** |  |  | Change | IUCN | Change | IUCN | Change | IUCN | Change | IUCN | Change | IUCN | Change | IUCN | Change | IUCN | Change | IUCN |
| *Capsicum flexuosum* | 353 | yes | -36.1 | VU | -47.7 | VU | -39.2 | VU | -60.1 | EN | -33.7 | VU | -35.8 | VU | -38.0 | VU | -57.1 | EN |
| *Pimenta pseudocaryophyllus* | 544 | yes | -45.3 | VU | -51.7 | EN | -52.9 | EN | -77.6 | EN | -38.9 | VU | -47.6 | VU | -44.8 | VU | -76.5 | EN |
| *Schinus terebinthifolia* | 1714 | yes | -28.6 |  | -33.6 | VU | -30.6 | VU | -47.0 | VU | -12.7 |  | -14.7 |  | -16.6 |  | -31.3 | VU |
| *Tropaeolum pentaphyllum* | 98 | yes | -30.9 | VU | -29.9 |  | -34.1 | VU | -39.2 | VU | -2.9 |  | 3.1 |  | 0.1 |  | -14.1 |  |
| ***Fibrous species*** |  |  |  |  |  |  |  |  |  |  |  |  |  |  |  |  |  |  |
| *Coleataenia prionitis* | 301 | yes | -17.9 |  | -38.5 | VU | -33.6 | VU | -52.1 | EN | 50.6 |  | 26.1 |  | 54.4 |  | 32.7 |  |
| *Geonoma gamiova* | 114 | yes | -6.1 |  | -6.6 |  | -17.5 |  | -35.2 | VU | 56.1 |  | 86.1 |  | 25.0 |  | 28.8 |  |
| *Gynerium sagittatum* | 363 | yes | -43.5 | VU | -47.8 | VU | -48.1 | VU | -58.8 | EN | -32.0 | VU | -34.2 | VU | -34.8 | VU | -42.0 | VU |
| *Philodendron corcovadense* | 53 | yes | -35.4 | VU | -38.1 | VU | -40.9 | VU | -45.1 | VU | -7.6 |  | 1.1 |  | -12.5 |  | 14.5 |  |
| *Schoenoplectus californicus* | 414 | yes | -27.5 |  | -32.8 | VU | -32.4 | VU | -51.0 | EN | -22.8 |  | -28.4 |  | -25.9 |  | -47.9 | VU |
| ***Food species*** |  |  |  |  |  |  |  |  |  |  |  |  |  |  |  |  |  |  |
| *Acca sellowiana* | 262 | yes | -22.6 |  | -28.7 |  | -34.6 | VU | -39.4 | VU | -7.1 |  | -20.1 |  | -10.3 |  | -13.4 |  |
| *Annona crassiflora* | 318 | yes | -68.2 | EN | -82.9 | CR | -76.3 | EN | -91.8 | CR | -61.0 | EN | -77.9 | EN | -71.5 | EN | -89.1 | CR |
| *Araucaria angustifolia* | 226 | yes | -46.2 | VU | -52.9 | EN | -52.7 | EN | -66.6 | EN | -46.1 | VU | -52.9 | EN | -52.6 | EN | -66.5 | EN |
| *Butia catarinensis* | 37 | no | 0.0 |  | 0.0 |  | 0.0 |  | 0.0 |  | 0.0 |  | 0.0 |  | 0.0 |  | 0.0 |  |
| *Butia eriospatha* | 38 | yes | -67.7 | EN | -77.3 | EN | -67.8 | EN | -87.4 | CR | -45.2 | VU | -50.4 | EN | -52.7 | EN | -64.3 | EN |
| *Campomanesia xanthocarpa* | 579 | yes | -38.9 | VU | -32.1 | VU | -36.6 | VU | -47.8 | VU | -35.5 | VU | -25.8 |  | -35.2 | VU | -43.3 | VU |
| *Eugenia involucrata* | 599 | yes | -42.4 | VU | -44.5 | VU | -45.6 | VU | -61.8 | EN | -39.9 | VU | -40.4 | VU | -43.6 | VU | -59.7 | EN |
| *Eugenia pyriformis* | 600 | yes | -34.2 | VU | -60.1 | EN | -53.4 | EN | -60.9 | EN | -17.6 |  | -38.9 | VU | -42.1 | VU | -48.8 | VU |
| *Eugenia uniflora* | 381 | yes | -39.4 | VU | -43.8 | VU | -42.8 | VU | -56.2 | EN | -9.6 |  | -8.0 |  | -16.6 |  | -33.8 | VU |
| *Euterpe edulis* | 392 | yes | -50.1 | EN | -52.7 | EN | -58.0 | EN | -69.7 | EN | -19.9 |  | -19.9 |  | -20.3 |  | -33.7 | VU |
| *Opuntia elata* | 58 | yes | -12.0 |  | -58.6 | EN | -57.8 | EN | -47.9 | VU | 45.8 |  | -28.7 |  | -26.2 |  | 12.3 |  |
| *Passiflora actinia* | 119 | yes | -46.5 | VU | -44.3 | VU | -41.6 | VU | -49.4 | VU | -37.3 | VU | -21.6 |  | -33.6 | VU | -26.2 |  |
| *Physalis pubescens* | 588 | yes | -43.2 | VU | -48.1 | VU | -47.1 | VU | -61.9 | EN | -41.4 | VU | -46.0 | VU | -45.5 | VU | -59.2 | EN |
| *Plinia peruviana* | 48 | yes | -79.6 | EN | -87.3 | CR | -76.0 | EN | -88.3 | CR | -56.9 | EN | -68.0 | EN | -60.2 | EN | -64.2 | EN |
| *Psidium cattleianum* | 576 | yes | -26.5 |  | -31.9 | VU | -37.4 | VU | -46.1 | VU | -10.2 |  | -20.7 |  | -26.6 |  | -34.0 | VU |
| *Vasconcellea quercifolia* | 600 | yes | -42.1 | VU | -51.5 | EN | -52.8 | EN | -50.3 | EN | -26.3 |  | -43.0 | VU | -44.5 | VU | -34.8 | VU |
| ***Forage species (Fabaceae)*** |  |  |  |  |  |  |  |  |  |  |  |  |  |  |  |  |  |  |
| *Adesmia bicolor* | 98 | yes | -64.5 | EN | -69.0 | EN | -74.1 | EN | -80.1 | CR | -48.2 | VU | -58.5 | EN | -48.6 | VU | -76.0 | EN |
| *Adesmia latifolia* | 38 | yes | -68.3 | EN | -47.2 | VU | -51.8 | EN | -52.9 | EN | 40.8 |  | 39.3 |  | 13.8 |  | 23.5 |  |
| *Adesmia securigerifolia* | 27 | no | 0.0 |  | 0.0 |  | 0.0 |  | 0.0 |  | 0.0 |  | 0.0 |  | 0.0 |  | 0.0 |  |
| *Adesmia tristis* | 51 | yes | -94.4 | CR | -97.0 | CR | -98.0 | CR | -99.2 | CR | -94.4 | CR | -96.9 | CR | -98.0 | CR | -99.2 | CR |
| *Desmodium adscendens* | 1282 | yes | -15.7 |  | -14.4 |  | -18.5 |  | -16.3 |  | 76.6 |  | 122.9 |  | 91.9 |  | 190.9 |  |
| *Desmodium barbatum* | 1708 | yes | -17.0 |  | -17.5 |  | -19.8 |  | -24.1 |  | 25.0 |  | 29.0 |  | 30.8 |  | 29.0 |  |
| *Desmodium incanum* | 1627 | yes | -27.7 |  | -29.9 |  | -31.9 | VU | -41.9 | VU | -21.5 |  | -24.5 |  | -27.6 |  | -37.7 | VU |
| *Desmodium subsericeum* | 137 | yes | -59.1 | EN | -70.8 | EN | -65.1 | EN | -77.5 | EN | -44.4 | VU | -63.7 | EN | -55.7 | EN | -68.7 | EN |
| *Indigofera sabulicola* | 80 | yes | 0.3 |  | 0.3 |  | 0.5 |  | 0.0 |  | 183.7 |  | 218.4 |  | 191.0 |  | 387.7 |  |
| *Leptospron adenanthum* | 477 | yes | -10.5 |  | -11.8 |  | -12.6 |  | -10.3 |  | 62.7 |  | 79.6 |  | 74.6 |  | 119.2 |  |
| *Macroptilium psammodes* | 47 | yes | -20.9 |  | -30.3 | VU | -32.3 | VU | -12.9 |  | 115.3 |  | 123.1 |  | 115.5 |  | 227.9 |  |
| *Ornithopus micranthus* | 12 | yes | -100.0 | CR | -100.0 | CR | -100.0 | CR | -95.1 | CR | -100.0 | CR | -100.0 | CR | -95.1 | CR | -95.1 | CR |
| *Stylosanthes leiocarpa* | 105 | yes | -37.6 | VU | -36.2 | VU | -37.2 | VU | -30.7 | VU | -21.8 |  | -2.5 |  | -14.8 |  | 19.4 |  |
| *Trifolium polymorphum* | 103 | yes | -47.1 | VU | -48.8 | VU | -67.6 | EN | -81.8 | CR | 2.6 |  | 2.3 |  | -5.0 |  | -48.1 | VU |
| *Trifolium riograndense* | 29 | yes | -91.1 | CR | -75.5 | EN | -95.3 | CR | -86.4 | CR | -91.1 | CR | -75.2 | EN | -95.3 | CR | -86.4 | CR |
| *Vigna luteola* | 515 | yes | -16.9 |  | -23.1 |  | -21.7 |  | -26.2 |  | 0.5 |  | -4.7 |  | -5.8 |  | -11.8 |  |
| ***Forage species (Poaceae)*** |  |  |  |  |  |  |  |  |  |  |  |  |  |  |  |  |  |  |
| *Axonopus compressus* | 1022 | yes | -36.4 | VU | -34.8 | VU | -37.5 | VU | -55.2 | EN | -23.8 |  | -19.7 |  | -22.6 |  | -42.0 | VU |
| *Axonopus fissifolius* | 588 | yes | -24.6 |  | -26.5 |  | -28.0 |  | -34.0 | VU | 98.5 |  | 143.3 |  | 121.8 |  | 182.7 |  |
| *Axonopus obtusifolius* | 61 | yes | -53.4 | EN | -57.9 | EN | -59.1 | EN | -76.0 | EN | 0.2 |  | -3.6 |  | -7.1 |  | -34.3 | VU |
| *Bothriochloa laguroides* | 214 | yes | -54.8 | EN | -50.9 | EN | -60.6 | EN | -77.3 | EN | -38.6 | VU | -23.8 |  | -37.6 | VU | -65.5 | EN |
| *Bromus auleticus* | 165 | yes | -81.0 | CR | -82.3 | CR | -69.7 | EN | -88.5 | CR | -79.2 | EN | -80.3 | CR | -52.3 | EN | -83.6 | CR |
| *Bromus catharticus* | 597 | yes | -35.8 | VU | -44.3 | VU | -40.7 | VU | -49.6 | VU | -33.2 | VU | -41.5 | VU | -36.2 | VU | -45.8 | VU |
| *Dichanthelium sabulorum* | 103 | yes | -35.4 | VU | -38.2 | VU | -45.1 | VU | -62.9 | EN | -34.4 | VU | -33.9 | VU | -44.0 | VU | -60.3 | EN |
| *Echinochloa polystachya* | 499 | yes | -7.6 |  | -8.3 |  | -9.8 |  | -10.8 |  | 174.3 |  | 210.0 |  | 187.5 |  | 259.4 |  |
| *Hemarthria altissima* | 149 | yes | -11.4 |  | -7.0 |  | -4.6 |  | -8.4 |  | 60.2 |  | 47.2 |  | 73.2 |  | 116.0 |  |
| *Ischaemum minus* | 37 | yes | -29.9 |  | -40.9 | VU | -47.1 | VU | -6.0 |  | 42.2 |  | 36.8 |  | 22.6 |  | 141.5 |  |
| *Mnesithea selloana* | 76 | yes | -41.1 | VU | -49.9 | VU | -64.4 | EN | -72.4 | EN | 36.3 |  | 28.6 |  | 30.0 |  | 23.3 |  |
| *Nassella neesiana* | 503 | yes | -30.7 | VU | -31.9 | VU | -34.4 | VU | -46.3 | VU | -26.3 |  | -29.4 |  | -21.2 |  | -45.4 | VU |
| *Paspalum almum* | 178 | yes | -2.0 |  | -18.8 |  | -14.4 |  | -16.9 |  | 93.1 |  | 48.4 |  | 54.3 |  | 63.5 |  |
| *Paspalum denticulatum* | 261 | yes | -22.9 |  | -31.1 | VU | -31.2 | VU | -43.5 | VU | -1.8 |  | 0.6 |  | 7.8 |  | -17.9 |  |
| *Paspalum dilatatum* | 335 | yes | -26.1 |  | -26.3 |  | -27.8 |  | -45.8 | VU | -25.6 |  | -26.0 |  | -27.2 |  | -45.7 | VU |
| *Paspalum glaucescens* | 161 | yes | -59.0 | EN | -42.0 | VU | -55.2 | EN | -61.0 | EN | -36.7 | VU | 5.7 |  | -37.8 | VU | -41.9 | VU |
| *Paspalum guenoarum* | 205 | yes | -24.1 |  | -28.3 |  | -31.4 | VU | -42.3 | VU | -11.9 |  | -14.6 |  | -17.8 |  | -28.1 |  |
| *Paspalum jesuiticum* | 38 | yes | -69.4 | EN | -74.1 | EN | -81.2 | CR | -95.9 | CR | -69.4 | EN | -74.1 | EN | -81.2 | CR | -95.9 | CR |
| *Paspalum lepton* | 199 | yes | -5.6 |  | -6.6 |  | -12.3 |  | -5.4 |  | 117.6 |  | 111.5 |  | 137.9 |  | 127.1 |  |
| *Paspalum modestum* | 41 | yes | 0.3 |  | -4.1 |  | -1.6 |  | -4.2 |  | 110.6 |  | 99.8 |  | 102.6 |  | 143.0 |  |
| *Paspalum notatum* | 595 | yes | -26.0 |  | -27.1 |  | -28.0 |  | -42.0 | VU | -25.2 |  | -26.0 |  | -26.9 |  | -40.6 | VU |
| *Paspalum pumilum* | 195 | yes | -41.8 | VU | -39.3 | VU | -39.7 | VU | -50.2 | EN | -41.0 | VU | -34.7 | VU | -38.5 | VU | -47.9 | VU |
| *Paspalum regnellii* | 116 | yes | -16.8 |  | -37.4 | VU | -29.0 |  | -40.4 | VU | 40.4 |  | 17.0 |  | 7.5 |  | 20.1 |  |
| *Paspalum rhodopedum* | 30 | no | 0.0 |  | 0.0 |  | 0.0 |  | 0.0 |  | 0.0 |  | 0.0 |  | 0.0 |  | 0.0 |  |
| *Poa lanigera* | 159 | yes | -30.2 | VU | -28.6 |  | -30.3 | VU | -46.7 | VU | -3.2 |  | -9.1 |  | 4.1 |  | -25.3 |  |
| *Schizachyrium tenerum* | 428 | yes | -49.8 | VU | -51.2 | EN | -53.4 | EN | -68.9 | EN | -46.3 | VU | -46.2 | VU | -50.0 | EN | -64.3 | EN |
| ***Medicinal species*** |  |  |  |  |  |  |  |  |  |  |  |  |  |  |  |  |  |  |
| *Achyrocline satureioides* | 1352 | yes | -45.7 | VU | -48.0 | VU | -47.8 | VU | -59.1 | EN | -45.0 | VU | -47.0 | VU | -47.3 | VU | -57.4 | EN |
| *Baccharis articulata* | 512 | yes | -48.8 | VU | -53.2 | EN | -50.9 | EN | -58.7 | EN | -44.2 | VU | -43.7 | VU | -45.1 | VU | -53.3 | EN |
| *Baccharis crispa* | 589 | yes | -39.2 | VU | -45.4 | VU | -44.7 | VU | -57.8 | EN | -38.2 | VU | -42.7 | VU | -43.1 | VU | -56.6 | EN |
| *Baccharis dracunculifolia* | 1216 | yes | -42.9 | VU | -52.8 | EN | -51.6 | EN | -64.5 | EN | -37.4 | VU | -44.7 | VU | -45.6 | VU | -58.4 | EN |
| *Bauhinia forficata* | 1002 | yes | -42.5 | VU | -44.9 | VU | -43.4 | VU | -55.3 | EN | -33.0 | VU | -32.4 | VU | -33.1 | VU | -45.1 | VU |
| *Bromelia antiacantha* | 66 | yes | -28.2 |  | -29.1 |  | -26.0 |  | -23.4 |  | 26.5 |  | 34.6 |  | 26.9 |  | 27.0 |  |
| *Casearia sylvestris* | 5464 | yes | -37.1 | VU | -38.3 | VU | -39.0 | VU | -50.4 | EN | -7.3 |  | -7.5 |  | -6.8 |  | 1.7 |  |
| *Cecropia glaziovii* | 180 | yes | -42.6 | VU | -51.8 | EN | -55.4 | EN | -69.1 | EN | -21.7 |  | -41.0 | VU | -40.3 | VU | -54.2 | EN |
| *Copaifera trapezifolia* | 135 | yes | -19.3 |  | -22.3 |  | -25.1 |  | -39.6 | VU | 20.1 |  | 9.5 |  | 15.0 |  | -17.5 |  |
| *Croton celtidifolius* | 191 | yes | -35.0 | VU | -54.2 | EN | -44.0 | VU | -81.6 | CR | -32.8 | VU | -53.3 | EN | -40.6 | VU | -81.4 | CR |
| *Cunila microcephala* | 21 | yes | -100.0 | CR | -100.0 | CR | -100.0 | CR | -100.0 | CR | -42.4 | VU | -33.1 | VU | -48.3 | VU | -88.1 | CR |
| *Drimys brasiliensis* | 600 | yes | -73.8 | EN | -76.9 | EN | -77.9 | EN | -91.7 | CR | -73.8 | EN | -76.9 | EN | -77.9 | EN | -91.7 | CR |
| *Echinodorus grandiflorus* | 294 | yes | -20.0 |  | -28.5 |  | -26.8 |  | -36.5 | VU | -17.4 |  | -23.9 |  | -23.3 |  | -31.4 | VU |
| *Equisetum giganteum* | 595 | yes | -36.2 | VU | -39.8 | VU | -38.1 | VU | -48.7 | VU | -31.0 | VU | -34.8 | VU | -31.8 | VU | -40.7 | VU |
| *Hypericum caprifoliatum* | 83 | yes | -5.5 |  | -11.2 |  | -12.8 |  | -19.9 |  | 29.6 |  | 7.2 |  | 2.6 |  | 5.1 |  |
| *Ilex paraguariensis* | 1040 | yes | -40.0 | VU | -41.2 | VU | -46.3 | VU | -57.8 | EN | -38.8 | VU | -37.3 | VU | -44.9 | VU | -57.6 | EN |
| *Jodina rhombifolia* | 83 | yes | -57.5 | EN | -58.4 | EN | -59.5 | EN | -62.2 | EN | -51.5 | EN | -52.5 | EN | -53.0 | EN | -50.9 | EN |
| *Mikania glomerata* | 285 | yes | -41.7 | VU | -37.1 | VU | -47.6 | VU | -56.9 | EN | -12.9 |  | -6.0 |  | -18.8 |  | -24.4 |  |
| *Mikania laevigata* | 161 | yes | -27.4 |  | -23.0 |  | -26.5 |  | -32.8 | VU | -12.8 |  | 3.2 |  | -14.4 |  | -4.5 |  |
| *Monteverdia ilicifolia* | 500 | yes | -23.7 |  | -36.8 | VU | -37.5 | VU | -41.5 | VU | -18.9 |  | -30.5 | VU | -33.2 | VU | -36.5 | VU |
| *Ocimum carnosum* | 570 | yes | -17.3 |  | -41.1 | VU | -36.1 | VU | -35.7 | VU | -10.3 |  | -34.4 | VU | -30.0 | VU | -29.4 |  |
| *Piper umbellatum* | 595 | yes | -43.7 | VU | -50.4 | EN | -48.9 | VU | -63.7 | EN | -37.6 | VU | -42.2 | VU | -45.0 | VU | -54.4 | EN |
| *Plantago australis* | 1047 | yes | -38.5 | VU | -42.0 | VU | -40.6 | VU | -53.3 | EN | -34.0 | VU | -37.3 | VU | -35.1 | VU | -46.1 | VU |
| *Sambucus australis* | 180 | yes | -32.5 | VU | -37.8 | VU | -38.0 | VU | -39.5 | VU | -26.3 |  | -34.1 | VU | -29.6 |  | -32.7 | VU |
| *Smilax campestris* | 554 | yes | -44.7 | VU | -45.7 | VU | -47.0 | VU | -62.2 | EN | -37.6 | VU | -36.2 | VU | -41.4 | VU | -54.9 | EN |
| *Solanum mauritianum* | 523 | yes | -43.5 | VU | -45.2 | VU | -48.8 | VU | -61.0 | EN | -39.1 | VU | -39.2 | VU | -45.9 | VU | -58.3 | EN |
| *Solanum paniculatum* | 1898 | yes | -42.6 | VU | -54.0 | EN | -46.9 | VU | -62.0 | EN | -26.4 |  | -38.2 | VU | -30.5 | VU | -30.1 | VU |
| *Sorocea bonplandii* | 600 | yes | -43.8 | VU | -56.4 | EN | -52.3 | EN | -68.1 | EN | -21.1 |  | -39.5 | VU | -21.9 |  | -58.3 | EN |
| *Trichilia catigua* | 1077 | yes | -38.5 | VU | -66.0 | EN | -56.5 | EN | -79.4 | EN | -8.5 |  | -34.9 | VU | -23.5 |  | -51.8 | EN |
| *Varronia curassavica* | 1684 | yes | -29.1 |  | -33.2 | VU | -32.5 | VU | -36.5 | VU | 65.3 |  | 122.4 |  | 83.6 |  | 229.5 |  |
| *Wilbrandia ebracteata* | 151 | yes | -36.3 | VU | -28.6 |  | -23.2 |  | -60.5 | EN | 28.2 |  | 20.5 |  | 42.3 |  | -33.6 | VU |
| *Zollernia ilicifolia* | 235 | yes | -38.8 | VU | -42.5 | VU | -42.4 | VU | -57.4 | EN | -14.1 |  | -6.6 |  | -15.0 |  | -36.1 | VU |
| ***Ornamental species*** |  |  |  |  |  |  |  |  |  |  |  |  |  |  |  |  |  |  |
| *Ananas bracteatus* | 67 | yes | -44.6 | VU | -46.6 | VU | -46.0 | VU | -60.2 | EN | -37.3 | VU | -37.7 | VU | -39.5 | VU | -52.7 | EN |
| *Aspilia montevidensis* | 276 | yes | -27.5 |  | -28.9 |  | -35.6 | VU | -27.6 |  | -2.3 |  | 9.9 |  | -10.8 |  | 13.4 |  |
| *Calliandra tweedii* | 221 | yes | -32.7 | VU | -42.9 | VU | -42.4 | VU | -52.5 | EN | -27.8 |  | -33.9 | VU | -38.1 | VU | -44.5 | VU |
| *Cortaderia selloana* | 257 | yes | -35.0 | VU | -44.0 | VU | -39.2 | VU | -57.7 | EN | -30.1 | VU | -39.4 | VU | -31.6 | VU | -52.6 | EN |
| *Dyckia distachya* | 43 | yes | -44.7 | VU | -99.6 | CR | -71.8 | EN | -91.7 | CR | 159.0 |  | -36.8 | VU | 44.4 |  | 21.4 |  |
| *Epidendrum fulgens* | 85 | yes | -18.1 |  | -19.0 |  | -37.5 | VU | -3.7 |  | 30.3 |  | 123.1 |  | 14.2 |  | 264.0 |  |
| *Fuchsia regia* | 499 | yes | -51.2 | EN | -56.4 | EN | -56.9 | EN | -79.9 | EN | -49.7 | VU | -54.9 | EN | -55.6 | EN | -79.3 | EN |
| *Gomesa flexuosa* | 179 | yes | -38.1 | VU | -40.6 | VU | -39.2 | VU | -60.5 | EN | -23.0 |  | -31.4 | VU | -24.6 |  | -52.4 | EN |
| *Handroanthus chrysotrichus* | 307 | yes | -38.5 | VU | -43.2 | VU | -45.3 | VU | -60.5 | EN | -14.9 |  | -25.0 |  | -21.0 |  | -40.7 | VU |
| *Heliconia farinosa* | 199 | yes | -13.7 |  | -15.5 |  | -12.6 |  | -20.6 |  | 27.7 |  | 64.1 |  | 71.9 |  | 29.1 |  |
| *Jacaranda puberula* | 506 | yes | -40.2 | VU | -49.4 | VU | -50.1 | EN | -63.7 | EN | -27.0 |  | -36.9 | VU | -36.2 | VU | -52.5 | EN |
| *Parodia ottonis* | 149 | yes | -63.9 | EN | -56.0 | EN | -72.6 | EN | -82.5 | CR | -51.8 | EN | -42.4 | VU | -68.5 | EN | -76.6 | EN |
| *Petunia integrifolia* | 361 | yes | -4.2 |  | -5.6 |  | -12.7 |  | -12.2 |  | 7.3 |  | 1.2 |  | -10.1 |  | -4.9 |  |
| *Pyrostegia venusta* | 1523 | yes | -49.3 | VU | -46.6 | VU | -46.3 | VU | -48.9 | VU | -12.0 |  | 22.2 |  | 8.4 |  | 70.5 |  |
| *Rumohra adiantiformis* | 576 | yes | -34.9 | VU | -38.3 | VU | -41.6 | VU | -52.2 | EN | -34.6 | VU | -36.6 | VU | -41.5 | VU | -51.8 | EN |
| *Sinningia leucotricha* | 14 | no | 0.0 |  | 0.0 |  | 0.0 |  | 0.0 |  | 0.0 |  | 0.0 |  | 0.0 |  | 0.0 |  |
| *Syagrus romanzoffiana* | 229 | yes | -61.0 | EN | -62.4 | EN | -61.8 | EN | -75.5 | EN | -27.3 |  | -30.0 | VU | -21.7 |  | -56.0 | EN |
| *Tibouchina sellowiana* | 365 | yes | -41.8 | VU | -53.9 | EN | -50.3 | EN | -74.5 | EN | -9.1 |  | -39.6 | VU | -35.0 | VU | -69.8 | EN |
| *Trichocline catharinensis* | 127 | yes | -54.1 | EN | -85.6 | CR | -83.3 | CR | -89.0 | CR | -53.0 | EN | -71.2 | EN | -83.3 | CR | -86.7 | CR |
| *Verbena rigida* | 381 | yes | -47.5 | VU | -68.4 | EN | -67.0 | EN | -69.3 | EN | -32.6 | VU | -50.8 | EN | -49.8 | VU | -66.2 | EN |
| ***Timber species*** |  |  |  |  |  |  |  |  |  |  |  |  |  |  |  |  |  |  |
| *Apuleia leiocarpa* | 923 | yes | -56.0 | EN | -61.0 | EN | -60.6 | EN | -75.3 | EN | -52.8 | EN | -57.0 | EN | -57.8 | EN | -71.1 | EN |
| *Aspidosperma polyneuron* | 599 | yes | -49.7 | VU | -57.1 | EN | -47.7 | VU | -63.6 | EN | 34.7 |  | 41.4 |  | 29.4 |  | 155.9 |  |
| *Ateleia glazioveana* | 92 | yes | -28.3 |  | -15.3 |  | -36.7 | VU | -17.0 |  | 17.3 |  | 39.2 |  | -11.5 |  | 27.4 |  |
| *Balfourodendron riedelianum* | 342 | yes | -29.0 |  | -52.7 | EN | -37.7 | VU | -51.6 | EN | -24.2 |  | -47.3 | VU | -31.1 | VU | -46.0 | VU |
| *Cabralea canjerana* | 1306 | yes | -42.4 | VU | -52.0 | EN | -50.9 | EN | -61.6 | EN | -40.4 | VU | -48.1 | VU | -48.5 | VU | -57.1 | EN |
| *Calophyllum brasiliense* | 1633 | yes | -39.8 | VU | -41.2 | VU | -43.3 | VU | -40.7 | VU | 104.5 |  | 137.5 |  | 125.3 |  | 183.0 |  |
| *Cedrela fissilis* | 935 | yes | -48.4 | VU | -54.1 | EN | -52.8 | EN | -67.1 | EN | -47.8 | VU | -52.6 | EN | -52.5 | EN | -66.3 | EN |
| *Colubrina glandulosa* | 364 | yes | -60.7 | EN | -59.6 | EN | -54.9 | EN | -74.7 | EN | -52.3 | EN | -49.9 | VU | -45.3 | VU | -69.8 | EN |
| *Cordia trichotoma* | 975 | yes | -54.0 | EN | -59.9 | EN | -58.4 | EN | -72.6 | EN | -41.5 | VU | -45.2 | VU | -44.8 | VU | -57.6 | EN |
| *Enterolobium contortisiliquum* | 598 | yes | -46.8 | VU | -63.3 | EN | -55.7 | EN | -74.8 | EN | -23.9 |  | -44.9 | VU | -32.0 | VU | -49.3 | VU |
| *Handroanthus heptaphyllus* | 389 | yes | -26.4 |  | -30.7 | VU | -38.3 | VU | -58.2 | EN | 28.0 |  | 25.7 |  | 7.3 |  | -12.4 |  |
| *Hyeronima alchorneoides* | 196 | yes | -34.8 | VU | -38.2 | VU | -36.3 | VU | -45.4 | VU | -1.1 |  | 13.7 |  | 9.7 |  | 32.6 |  |
| *Miconia cinnamomifolia* | 480 | yes | -8.9 |  | -15.5 |  | -14.6 |  | -27.0 |  | 27.9 |  | 12.4 |  | 15.0 |  | -13.0 |  |
| *Mimosa scabrella* | 318 | yes | -44.8 | VU | -61.5 | EN | -61.2 | EN | -83.5 | CR | -44.8 | VU | -61.4 | EN | -61.2 | EN | -83.5 | CR |
| *Nectandra lanceolata* | 542 | yes | -40.2 | VU | -52.1 | EN | -49.7 | VU | -60.8 | EN | -34.6 | VU | -47.3 | VU | -44.9 | VU | -56.9 | EN |
| *Ocotea puberula* | 1399 | yes | -38.2 | VU | -39.7 | VU | -39.7 | VU | -55.4 | EN | -34.0 | VU | -33.3 | VU | -35.4 | VU | -50.8 | EN |
| *Parapiptadenia rigida* | 594 | yes | -38.4 | VU | -48.3 | VU | -44.5 | VU | -56.5 | EN | -20.9 |  | -17.1 |  | -26.8 |  | -33.5 | VU |
| *Peltophorum dubium* | 596 | yes | -76.5 | EN | -79.4 | EN | -74.8 | EN | -88.4 | CR | -58.3 | EN | -61.5 | EN | -49.8 | VU | -68.9 | EN |
| *Piptocarpha angustifolia* | 71 | yes | -41.7 | VU | -65.7 | EN | -69.9 | EN | -72.2 | EN | -9.2 |  | -47.3 | VU | -59.5 | EN | -56.3 | EN |
| *Schizolobium parahyba* | 325 | yes | -38.6 | VU | -36.0 | VU | -36.7 | VU | -52.2 | EN | -24.1 |  | -11.1 |  | -15.9 |  | -39.1 | VU |
|  |  |  |  |  |  |  |  |  |  |  |  |  |  |  |  |  |  |  |
| Median range change (%) |  |  | -38.5 |  | -43.5 |  | -43.4 |  | -56.3 |  | -22.3 |  | -29.7 |  | -27.4 |  | -41.9 |  |

Table S2. Brazilian agroforestry plant species qualified for the IUCN threatened category (VU, EN, CR) based on B1 and B2 criteria.

| **Taxa** | **EOO** | **AOO** | **Nbe unique occ.** | **Nbe subPop** | **Nbe loc** | **Category CriteriaB** | **Category code** | **Category AOO** | **Category EOO** |
| --- | --- | --- | --- | --- | --- | --- | --- | --- | --- |
| *Acca sellowiana* | 6999702 | 344 | 86 | 86 | 86 | LC or NT | LC or NT B1a+B2a | LC or NT | LC or NT |
| *Achyrocline satureioides* | 14540778 | 1804 | 451 | 451 | 451 | LC or NT | LC or NT B1a+B2a | LC or NT | LC or NT |
| *Adesmia bicolor* | 787090 | 188 | 47 | 47 | 47 | LC or NT | LC or NT B1a+B2a | LC or NT | LC or NT |
| *Adesmia latifolia* | 674267 | 68 | 17 | 17 | 17 | LC or NT | LC or NT B1a+B2a | LC or NT | LC or NT |
| *Adesmia securigerifolia* | 25323 | 28 | 7 | 7 | 7 | VU | VU B2a | VU | LC or NT |
| *Adesmia tristis* | 179529 | 84 | 21 | 21 | 21 | LC or NT | LC or NT B1a+B2a | LC or NT | LC or NT |
| *Ananas bracteatus* | 8811171 | 180 | 45 | 45 | 45 | LC or NT | LC or NT B1a+B2a | LC or NT | LC or NT |
| *Annona crassiflora* | 2256136 | 544 | 136 | 136 | 136 | LC or NT | LC or NT B1a+B2a | LC or NT | LC or NT |
| *Apuleia leiocarpa* | 10179173 | 1260 | 315 | 315 | 315 | LC or NT | LC or NT B1a+B2a | LC or NT | LC or NT |
| *Araucaria angustifolia* | 898636 | 392 | 98 | 98 | 98 | LC or NT | LC or NT B1a+B2a | LC or NT | LC or NT |
| *Aspidosperma polyneuron* | 6873955 | 220 | 55 | 55 | 55 | LC or NT | LC or NT B1a+B2a | LC or NT | LC or NT |
| *Aspilia montevidensis* | 820791 | 404 | 101 | 101 | 101 | LC or NT | LC or NT B1a+B2a | LC or NT | LC or NT |
| *Ateleia glazioveana* | 703844 | 128 | 32 | 32 | 32 | LC or NT | LC or NT B1a+B2a | LC or NT | LC or NT |
| *Axonopus compressus* | 15420146 | 1416 | 354 | 354 | 354 | LC or NT | LC or NT B1a+B2a | LC or NT | LC or NT |
| *Axonopus fissifolius* | 15296979 | 1004 | 251 | 251 | 251 | LC or NT | LC or NT B1a+B2a | LC or NT | LC or NT |
| *Axonopus obtusifolius* | 1296345 | 104 | 26 | 26 | 26 | LC or NT | LC or NT B1a+B2a | LC or NT | LC or NT |
| *Baccharis articulata* | 2772473 | 776 | 194 | 194 | 194 | LC or NT | LC or NT B1a+B2a | LC or NT | LC or NT |
| *Baccharis crispa* | 3176200 | 884 | 221 | 221 | 221 | LC or NT | LC or NT B1a+B2a | LC or NT | LC or NT |
| *Baccharis dracunculifolia* | 3602603 | 1588 | 397 | 397 | 397 | LC or NT | LC or NT B1a+B2a | LC or NT | LC or NT |
| *Balfourodendron riedelianum* | 2831317 | 536 | 134 | 134 | 134 | LC or NT | LC or NT B1a+B2a | LC or NT | LC or NT |
| *Bauhinia forficata* | 5631869 | 1460 | 365 | 365 | 365 | LC or NT | LC or NT B1a+B2a | LC or NT | LC or NT |
| *Bothriochloa laguroides* | 1540363 | 392 | 98 | 98 | 98 | LC or NT | LC or NT B1a+B2a | LC or NT | LC or NT |
| *Bromelia antiacantha* | 1058265 | 172 | 43 | 43 | 43 | LC or NT | LC or NT B1a+B2a | LC or NT | LC or NT |
| *Bromus auleticus* | 1427700 | 296 | 74 | 74 | 74 | LC or NT | LC or NT B1a+B2a | LC or NT | LC or NT |
| *Bromus catharticus* | 10922335 | 960 | 240 | 240 | 240 | LC or NT | LC or NT B1a+B2a | LC or NT | LC or NT |
| *Butia catarinensis* | 12054 | 32 | 8 | 8 | 8 | VU | VU B1a+B2a | VU | VU |
| *Butia eriospatha* | 271802 | 88 | 22 | 22 | 22 | LC or NT | LC or NT B1a+B2a | LC or NT | LC or NT |
| *Cabralea canjerana* | 7710590 | 1492 | 373 | 373 | 373 | LC or NT | LC or NT B1a+B2a | LC or NT | LC or NT |
| *Calliandra tweedii* | 2924153 | 396 | 99 | 99 | 99 | LC or NT | LC or NT B1a+B2a | LC or NT | LC or NT |
| *Calophyllum brasiliense* | 12376033 | 1992 | 498 | 498 | 498 | LC or NT | LC or NT B1a+B2a | LC or NT | LC or NT |
| *Campomanesia xanthocarpa* | 1676337 | 828 | 207 | 207 | 207 | LC or NT | LC or NT B1a+B2a | LC or NT | LC or NT |
| *Capsicum flexuosum* | 775394 | 456 | 114 | 114 | 114 | LC or NT | LC or NT B1a+B2a | LC or NT | LC or NT |
| *Casearia sylvestris* | 15478279 | 10136 | 3083 | 1554 | 2005 | LC or NT | LC or NT B1a+B2a | LC or NT | LC or NT |
| *Cecropia glaziovii* | 1035036 | 460 | 123 | 96 | 104 | LC or NT | LC or NT B1a+B2a | LC or NT | LC or NT |
| *Cedrela fissilis* | 7042650 | 2000 | 581 | 399 | 452 | LC or NT | LC or NT B1a+B2a | LC or NT | LC or NT |
| *Coleataenia prionitis* | 548358 | 324 | 81 | 81 | 81 | LC or NT | LC or NT B1a+B2a | LC or NT | LC or NT |
| *Colubrina glandulosa* | 11519147 | 836 | 249 | 158 | 180 | LC or NT | LC or NT B1a+B2a | LC or NT | LC or NT |
| *Copaifera trapezifolia* | 1917291 | 260 | 79 | 51 | 57 | LC or NT | LC or NT B1a+B2a | LC or NT | LC or NT |
| *Cordia trichotoma* | 4392647 | 2132 | 609 | 431 | 480 | LC or NT | LC or NT B1a+B2a | LC or NT | LC or NT |
| *Cortaderia selloana* | 10265083 | 524 | 131 | 131 | 131 | LC or NT | LC or NT B1a+B2a | LC or NT | LC or NT |
| *Croton celtidifolius* | 2252377 | 456 | 133 | 90 | 99 | LC or NT | LC or NT B1a+B2a | LC or NT | LC or NT |
| *Cunila microcephala* | 585428 | 56 | 14 | 14 | 14 | LC or NT | LC or NT B1a+B2a | LC or NT | LC or NT |
| *Desmodium adscendens* | 14067033 | 1880 | 470 | 470 | 470 | LC or NT | LC or NT B1a+B2a | LC or NT | LC or NT |
| *Desmodium barbatum* | 13795118 | 2584 | 646 | 646 | 646 | LC or NT | LC or NT B1a+B2a | LC or NT | LC or NT |
| *Desmodium incanum* | 16580945 | 2644 | 661 | 661 | 661 | LC or NT | LC or NT B1a+B2a | LC or NT | LC or NT |
| *Desmodium subsericeum* | 2297339 | 216 | 54 | 54 | 54 | LC or NT | LC or NT B1a+B2a | LC or NT | LC or NT |
| *Dichanthelium sabulorum* | 788312 | 196 | 49 | 49 | 49 | LC or NT | LC or NT B1a+B2a | LC or NT | LC or NT |
| *Drimys brasiliensis* | 1580594 | 1224 | 403 | 185 | 237 | LC or NT | LC or NT B1a+B2a | LC or NT | LC or NT |
| *Dyckia distachya* | 98578 | 52 | 13 | 13 | 13 | LC or NT | LC or NT B1a+B2a | LC or NT | LC or NT |
| *Echinochloa polystachya* | 15594791 | 784 | 196 | 196 | 196 | LC or NT | LC or NT B1a+B2a | LC or NT | LC or NT |
| *Echinodorus grandiflorus* | 12544057 | 636 | 159 | 159 | 159 | LC or NT | LC or NT B1a+B2a | LC or NT | LC or NT |
| *Enterolobium contortisiliquum* | 5091832 | 1560 | 446 | 312 | 345 | LC or NT | LC or NT B1a+B2a | LC or NT | LC or NT |
| *Epidendrum fulgens* | 102112 | 120 | 30 | 30 | 30 | LC or NT | LC or NT B1a+B2a | LC or NT | LC or NT |
| *Equisetum giganteum* | 11386476 | 1028 | 257 | 257 | 257 | LC or NT | LC or NT B1a+B2a | LC or NT | LC or NT |
| *Eugenia involucrata* | 3109792 | 1292 | 380 | 241 | 276 | LC or NT | LC or NT B1a+B2a | LC or NT | LC or NT |
| *Eugenia pyriformis* | 2316104 | 1304 | 384 | 235 | 277 | LC or NT | LC or NT B1a+B2a | LC or NT | LC or NT |
| *Eugenia uniflora* | 1145400 | 820 | 238 | 145 | 171 | LC or NT | LC or NT B1a+B2a | LC or NT | LC or NT |
| *Euterpe edulis* | 1964485 | 680 | 189 | 133 | 147 | LC or NT | LC or NT B1a+B2a | LC or NT | LC or NT |
| *Fuchsia regia* | 585461 | 472 | 118 | 118 | 118 | LC or NT | LC or NT B1a+B2a | LC or NT | LC or NT |
| *Geonoma gamiova* | 136172 | 188 | 47 | 47 | 47 | LC or NT | LC or NT B1a+B2a | LC or NT | LC or NT |
| *Gomesa flexuosa* | 1214029 | 292 | 73 | 73 | 73 | LC or NT | LC or NT B1a+B2a | LC or NT | LC or NT |
| *Gynerium sagittatum* | 10747783 | 624 | 156 | 156 | 156 | LC or NT | LC or NT B1a+B2a | LC or NT | LC or NT |
| *Handroanthus chrysotrichus* | 3589769 | 816 | 222 | 176 | 191 | LC or NT | LC or NT B1a+B2a | LC or NT | LC or NT |
| *Handroanthus heptaphyllus* | 5135659 | 780 | 217 | 167 | 182 | LC or NT | LC or NT B1a+B2a | LC or NT | LC or NT |
| *Heliconia farinosa* | 1205773 | 264 | 66 | 66 | 66 | LC or NT | LC or NT B1a+B2a | LC or NT | LC or NT |
| *Hemarthria altissima* | 3045731 | 300 | 75 | 75 | 75 | LC or NT | LC or NT B1a+B2a | LC or NT | LC or NT |
| *Hyeronima alchorneoides* | 11827263 | 504 | 132 | 109 | 116 | LC or NT | LC or NT B1a+B2a | LC or NT | LC or NT |
| *Hypericum caprifoliatum* | 764643 | 160 | 40 | 40 | 40 | LC or NT | LC or NT B1a+B2a | LC or NT | LC or NT |
| *Ilex paraguariensis* | 3090076 | 1764 | 571 | 298 | 364 | LC or NT | LC or NT B1a+B2a | LC or NT | LC or NT |
| *Indigofera sabulicola* | 4293727 | 204 | 51 | 51 | 51 | LC or NT | LC or NT B1a+B2a | LC or NT | LC or NT |
| *Ischaemum minus* | 193064 | 72 | 18 | 18 | 18 | LC or NT | LC or NT B1a+B2a | LC or NT | LC or NT |
| *Jacaranda puberula* | 2454118 | 1172 | 336 | 209 | 256 | LC or NT | LC or NT B1a+B2a | LC or NT | LC or NT |
| *Jodina rhombifolia* | 1913183 | 252 | 65 | 52 | 55 | LC or NT | LC or NT B1a+B2a | LC or NT | LC or NT |
| *Leptospron adenanthum* | 15795072 | 872 | 218 | 218 | 218 | LC or NT | LC or NT B1a+B2a | LC or NT | LC or NT |
| *Macroptilium psammodes* | 490046 | 100 | 25 | 25 | 25 | LC or NT | LC or NT B1a+B2a | LC or NT | LC or NT |
| *Miconia cinnamomifolia* | 823888 | 904 | 268 | 149 | 183 | LC or NT | LC or NT B1a+B2a | LC or NT | LC or NT |
| *Mikania glomerata* | 1891632 | 512 | 128 | 128 | 128 | LC or NT | LC or NT B1a+B2a | LC or NT | LC or NT |
| *Mikania laevigata* | 1284681 | 216 | 54 | 54 | 54 | LC or NT | LC or NT B1a+B2a | LC or NT | LC or NT |
| *Mimosa scabrella* | 1523596 | 736 | 210 | 127 | 155 | LC or NT | LC or NT B1a+B2a | LC or NT | LC or NT |
| *Mnesithea selloana* | 595586 | 184 | 46 | 46 | 46 | LC or NT | LC or NT B1a+B2a | LC or NT | LC or NT |
| *Monteverdia ilicifolia* | 3580366 | 744 | 186 | 186 | 186 | LC or NT | LC or NT B1a+B2a | LC or NT | LC or NT |
| *Nassella neesiana* | 1774875 | 640 | 160 | 160 | 160 | LC or NT | LC or NT B1a+B2a | LC or NT | LC or NT |
| *Nectandra lanceolata* | 1292743 | 1040 | 313 | 203 | 228 | LC or NT | LC or NT B1a+B2a | LC or NT | LC or NT |
| *Ocimum carnosum* | 3717620 | 828 | 207 | 207 | 207 | LC or NT | LC or NT B1a+B2a | LC or NT | LC or NT |
| *Ocotea puberula* | 20379903 | 2696 | 807 | 480 | 569 | LC or NT | LC or NT B1a+B2a | LC or NT | LC or NT |
| *Opuntia elata* | 1304359 | 116 | 29 | 29 | 29 | LC or NT | LC or NT B1a+B2a | LC or NT | LC or NT |
| *Ornithopus micranthus* | 65958 | 32 | 8 | 8 | 8 | VU | VU B2a | VU | LC or NT |
| *Parapiptadenia rigida* | 1077133 | 1288 | 369 | 212 | 268 | LC or NT | LC or NT B1a+B2a | LC or NT | LC or NT |
| *Parodia ottonis* | 399275 | 220 | 55 | 55 | 55 | LC or NT | LC or NT B1a+B2a | LC or NT | LC or NT |
| *Paspalum almum* | 616641 | 280 | 70 | 70 | 70 | LC or NT | LC or NT B1a+B2a | LC or NT | LC or NT |
| *Paspalum denticulatum* | 1133069 | 436 | 109 | 109 | 109 | LC or NT | LC or NT B1a+B2a | LC or NT | LC or NT |
| *Paspalum dilatatum* | 9400947 | 572 | 143 | 143 | 143 | LC or NT | LC or NT B1a+B2a | LC or NT | LC or NT |
| *Paspalum glaucescens* | 1613296 | 224 | 56 | 56 | 56 | LC or NT | LC or NT B1a+B2a | LC or NT | LC or NT |
| *Paspalum guenoarum* | 2954403 | 336 | 84 | 84 | 84 | LC or NT | LC or NT B1a+B2a | LC or NT | LC or NT |
| *Paspalum jesuiticum* | 249665 | 40 | 10 | 10 | 10 | VU | VU B2a | VU | LC or NT |
| *Paspalum lepton* | 953636 | 292 | 73 | 73 | 73 | LC or NT | LC or NT B1a+B2a | LC or NT | LC or NT |
| *Paspalum modestum* | 266713 | 92 | 23 | 23 | 23 | LC or NT | LC or NT B1a+B2a | LC or NT | LC or NT |
| *Paspalum notatum* | 12858516 | 992 | 248 | 248 | 248 | LC or NT | LC or NT B1a+B2a | LC or NT | LC or NT |
| *Paspalum pumilum* | 7360325 | 348 | 87 | 87 | 87 | LC or NT | LC or NT B1a+B2a | LC or NT | LC or NT |
| *Paspalum regnellii* | 709963 | 208 | 52 | 52 | 52 | LC or NT | LC or NT B1a+B2a | LC or NT | LC or NT |
| *Paspalum rhodopedum* | 69362 | 28 | 7 | 7 | 7 | VU | VU B2a | VU | LC or NT |
| *Passiflora actinia* | 292158 | 100 | 25 | 25 | 25 | LC or NT | LC or NT B1a+B2a | LC or NT | LC or NT |
| *Peltophorum dubium* | 2987264 | 1412 | 404 | 278 | 314 | LC or NT | LC or NT B1a+B2a | LC or NT | LC or NT |
| *Petunia integrifolia* | 2851832 | 580 | 145 | 145 | 145 | LC or NT | LC or NT B1a+B2a | LC or NT | LC or NT |
| *Philodendron corcovadense* | 479136 | 100 | 25 | 25 | 25 | LC or NT | LC or NT B1a+B2a | LC or NT | LC or NT |
| *Physalis pubescens* | 15466155 | 1248 | 312 | 312 | 312 | LC or NT | LC or NT B1a+B2a | LC or NT | LC or NT |
| *Pimenta pseudocaryophyllus* | 2031989 | 900 | 302 | 137 | 179 | LC or NT | LC or NT B1a+B2a | LC or NT | LC or NT |
| *Piper umbellatum* | 13265963 | 1004 | 251 | 251 | 251 | LC or NT | LC or NT B1a+B2a | LC or NT | LC or NT |
| *Piptocarpha angustifolia* | 307432 | 180 | 50 | 42 | 44 | LC or NT | LC or NT B1a+B2a | LC or NT | LC or NT |
| *Plantago australis* | 12035189 | 1340 | 335 | 335 | 335 | LC or NT | LC or NT B1a+B2a | LC or NT | LC or NT |
| *Plinia peruviana* | 1275400 | 96 | 27 | 24 | 24 | LC or NT | LC or NT B1a+B2a | LC or NT | LC or NT |
| *Poa lanigera* | 1139317 | 232 | 58 | 58 | 58 | LC or NT | LC or NT B1a+B2a | LC or NT | LC or NT |
| *Psidium cattleianum* | 1750604 | 1340 | 397 | 214 | 274 | LC or NT | LC or NT B1a+B2a | LC or NT | LC or NT |
| *Pyrostegia venusta* | 15031676 | 2264 | 566 | 566 | 566 | LC or NT | LC or NT B1a+B2a | LC or NT | LC or NT |
| *Rumohra adiantiformis* | 4274817 | 816 | 204 | 204 | 204 | LC or NT | LC or NT B1a+B2a | LC or NT | LC or NT |
| *Sambucus australis* | 3500344 | 488 | 140 | 107 | 113 | LC or NT | LC or NT B1a+B2a | LC or NT | LC or NT |
| *Schinus terebinthifolia* | 2725935 | 3524 | 1036 | 538 | 699 | LC or NT | LC or NT B1a+B2a | LC or NT | LC or NT |
| *Schizachyrium tenerum* | 11748537 | 612 | 153 | 153 | 153 | LC or NT | LC or NT B1a+B2a | LC or NT | LC or NT |
| *Schizolobium parahyba* | 10915127 | 780 | 225 | 151 | 169 | LC or NT | LC or NT B1a+B2a | LC or NT | LC or NT |
| *Schoenoplectus californicus* | 10561705 | 576 | 144 | 144 | 144 | LC or NT | LC or NT B1a+B2a | LC or NT | LC or NT |
| *Sinningia leucotricha* | 28940 | 28 | 7 | 7 | 7 | VU | VU B2a | VU | LC or NT |
| *Smilax campestris* | 5785138 | 996 | 249 | 249 | 249 | LC or NT | LC or NT B1a+B2a | LC or NT | LC or NT |
| *Solanum mauritianum* | 3356506 | 1184 | 347 | 238 | 270 | LC or NT | LC or NT B1a+B2a | LC or NT | LC or NT |
| *Solanum paniculatum* | 4095690 | 1884 | 471 | 471 | 471 | LC or NT | LC or NT B1a+B2a | LC or NT | LC or NT |
| *Sorocea bonplandii* | 1456275 | 1060 | 299 | 191 | 220 | LC or NT | LC or NT B1a+B2a | LC or NT | LC or NT |
| *Stylosanthes leiocarpa* | 2248471 | 184 | 46 | 46 | 46 | LC or NT | LC or NT B1a+B2a | LC or NT | LC or NT |
| *Syagrus romanzoffiana* | 2341828 | 612 | 175 | 130 | 141 | LC or NT | LC or NT B1a+B2a | LC or NT | LC or NT |
| *Tibouchina sellowiana* | 536338 | 740 | 213 | 115 | 151 | LC or NT | LC or NT B1a+B2a | LC or NT | LC or NT |
| *Trichilia catigua* | 4287951 | 1844 | 576 | 299 | 373 | LC or NT | LC or NT B1a+B2a | LC or NT | LC or NT |
| *Trichocline catharinensis* | 137652 | 164 | 41 | 41 | 41 | LC or NT | LC or NT B1a+B2a | LC or NT | LC or NT |
| *Trifolium polymorphum* | 2331491 | 240 | 60 | 60 | 60 | LC or NT | LC or NT B1a+B2a | LC or NT | LC or NT |
| *Trifolium riograndense* | 105328 | 84 | 21 | 21 | 21 | LC or NT | LC or NT B1a+B2a | LC or NT | LC or NT |
| *Tropaeolum pentaphyllum* | 2351973 | 188 | 47 | 47 | 47 | LC or NT | LC or NT B1a+B2a | LC or NT | LC or NT |
| *Varronia curassavica* | 14436380 | 1980 | 495 | 495 | 495 | LC or NT | LC or NT B1a+B2a | LC or NT | LC or NT |
| *Vasconcellea quercifolia* | 2580512 | 1004 | 451 | 148 | 188 | LC or NT | LC or NT B1a+B2a | LC or NT | LC or NT |
| *Verbena rigida* | 1144300 | 512 | 128 | 128 | 128 | LC or NT | LC or NT B1a+B2a | LC or NT | LC or NT |
| *Vigna luteola* | 16680952 | 1020 | 255 | 255 | 255 | LC or NT | LC or NT B1a+B2a | LC or NT | LC or NT |
| *Wilbrandia ebracteata* | 560396 | 180 | 45 | 45 | 45 | LC or NT | LC or NT B1a+B2a | LC or NT | LC or NT |
| *Zollernia ilicifolia* | 2938759 | 520 | 148 | 109 | 120 | LC or NT | LC or NT B1a+B2a | LC or NT | LC or NT |

Table S3. Taxonomic authorities and common names for the Brazilian agroforestry plant species prioritized by the Brazilian Ministry of the Environment initiative Native species of the Brazilian flora of current and potential economic value - Plants for the Future - Southern Region.

| **Aromatic species** | **Common names** |
| --- | --- |
| *Capsicum flexuosum* Sendtn. | Pimenta-braba, pimenta-do-mato, pimenta-silvestre, pimenta-do-morro. |
| *Pimenta pseudocaryophillus* (Gomes) Landrum. | Cravo, pau-cravo, craveiro, louro-cravo, louro, craveiro-do-mato, chá-de-bugre. |
| *Schinus terebinthifolius* Raddi. | Aroeira-pimenteira, aroeira-vermelha, aroeira-mansa, aroeira, aroeira-braba, aroeira-branca, aroeira-da-praia, aroeira-do-brejo, aroeira-do-campo, aroeira-de-sabiá, aroeirinha, coração-de-bugre, fruto-de-sabiá, fruto-de-raposa, fruto-de-cutia, araguaraíba, corneíba, árvore-da-pimenta, cabuí, cambuí, lentisco. |
| *Tropaeolum pentaphyllum* Lam. | Crem, batata-crem, crem-de-baraço, crem-trepador, capuchinha, carrapicho, chagas, cinco-chagas, chagas-da-miúda, sapatinho-de-iaiá, sapatinho-do-diabo. |
| **Fibrous species** |  |
| *Coleataenia prionitis* (Nees) Soreng | Capim-santa-fé, palha-santa-fé, santa-fé, pii-guiycé, paja-brava |
| *Geonoma gamiova* Barb. Rodr. | Guaricana-de-folha-larga, ouricana, gamiova, palheira, palheira-de-folha-larga, aricana, uricana |
| *Gynerium sagittatum* (Aubl.) P.Beauv. | Cana-brava, ubá, cana-ubá, parimá, ariná, eguará, eraí, flecha, cana-flecha, flecha-de-urubu, cana-do-rio, canarana, canarana-flecha, cana-amarga, capim-uva, cana-selvagem |
| *Philodendron concovadense* Kunth | Cipó-imbé, cipó-preto |
| *Schoenoplectus californicus* (C.a.Mey.) Soják | Junco, junco-gigante, piri, tiririca-agulha |
| **Food species** |  |
| *Acca sellowiana* (Berg) Burret | Goiaba serrana, feijoa, goiaba-da-serra, goiaba-verde, pineaple-guava. |
| *Annona crassiflora* Mart. | Marolo, araticum-do-cerrado. |
| *Araucaria angustifolia* (Bertol.) Kuntze. | Pinheiro, araucária, pinheiro-do-paraná, pinheiro-brasileiro. |
| *Butia catarinensis* Noblick & Lorenzi | Butiá, butiá-cabeçudo, butiá-da-praia, butiazeiro, coquinho-azedo. |
| *Butia eriospatha* (Mart. Ex Drude) Becc. | Butiá-da-serra, butiá-serrano, butiazeiro-serrano, butiá-vermelho, butiá-peludo. |
| *Campomanesia xanthocarpa* (Mart.) O.Berg | Guabiroba, gabiroba, guabirobeira. |
| *Eugenia involucrata* Dc. | Cerejeira-do-rio-grande, cerejeira-do-mato, cereja-do-rio-grande, cereja-do-mato. |
| *Eugenia pyriformis* Cambess. | Uvaia, uvalha, ubaia, uvaeira, azedinha, pome-azedo. |
| *Eugenia uniflora* L. | Pitanga, pitangueira, pitanga-mulata, pitanga-preta. |
| *Euterpe edulis* Mart. | Juçara, palmito, palmito-juçara, ripeiro, açaí-da-mata-atlântica. |
| *Opuntia elata* Salm-Dyck | Arumbeva, arumbé, palmatória, cardo-palmatório, palma. |
| *Passiflora actinia* Hook. | Maracujá-do-mato, maracujá-silvestre, maracujá-redondinho. |
| *Physalis pubescens* L. | Fisális, joá-de-capote, juá-de-capote, tomate-de-capote, canapu, camapu, bucho-de-rã, bate-testa, juápoca, joá-poca, golden-berry. |
| *Plinia peruviana* (Poir.) Govaerts | Jabuticaba, jabuticabeira, brazilian-grape. |
| *Psidium cattleianum* Sabine | Araçá, araçá-vermelho, araçá-amarelo, araçazeiro, araçazeiro-da-praia. |
| *Vasconcellea quercifolia* A. St. –Hil. | Jaracatiá, mamãozinho, mamoeiro-do-mato, mamute, mamão-brabo, figo-de-índio, coco-de-podre. |
| **Forage species (Fabaceae)** |  |
| *Adesma bicolor* (Poir.) DC. |  |
| *Adesmia latifolia* (Spreng.) Vogel |  |
| *Adesmia securigerifolia* Herter |  |
| *Adesmia tristis* Vogel |  |
| *Desmodium adscendens* (Sw.) DC. |  |
| *Desmodium barbatum* (L.) Benth. |  |
| *Desmodium incanum DC.* |  |
| *Desmodium subsericeum* Malme |  |
| *Indigofera sabulicola* Benth. |  |
| *Leptospron adenanthum* (G.Mey.) A.Delgado |  |
| *Macroptilium psammodes* (Lindm.) S.I. Drewes & R.A. Palacios. |  |
| *Ornithopus micranthus* (Benth.) Arechav. |  |
| *Stylosanthes leiocarpa* Vogel. |  |
| *Trifolium polymorphum* Poir. |  |
| *Trifolium rio-grandense* Burkart |  |
| *Vigna luteola* (JAcq.) Benth. |  |
| **Forage species (Poaceae)** |  |
| *Axonopus compressus* (Sw.) P. Beauv. |  |
| *Axonopus fissifolius* (Raddi) Kuhlm. |  |
| *Axonopus obtusifolius* (Raddi) Chase |  |
| *Bothriochloa laguroides* (DC.) Herter |  |
| *Bromus auleticus* Trin. Ex Nees |  |
| *Bromus catharticus* Vahl. |  |
| *Dichanthelium sabulorum* (Lam.) Gould & C.A. Clark |  |
| *Echinochloa polystachya* (kunth) Hitchc. |  |
| *Hemarthria altissima* (Poir.) Stapf & C.E.Hubb |  |
| *Ischaemum minus* J.Presl |  |
| *Mnesithea selloana* (Hack.) de Koning & Sosef |  |
| *Nassella neesiana* (Trin. & Rupr.) Barkworth |  |
| *Paspalum almum* Chase |  |
| *Paspalum denticulatum* Trin. |  |
| *Paspalum dilatatum* Poir. |  |
| *Paspalum glaucescens* Hack. |  |
| *Paspalum guenoarum* Arechav. |  |
| *Paspalum jesuiticum* Parodi |  |
| *Paspalum lepton* Schult. |  |
| *Paspalum modestum* Mez |  |
| *Paspalum notatum* Fluggé |  |
| *Paspalum pumilum* Nees |  |
| *Paspalum regnellii* Mez |  |
| *Paspalum rhodopedum* L.B.Sm. & Wassh. |  |
| *Poa lanigera* Nees |  |
| *Schizachyrium tenerum* Nees |  |
| **Medicinal species** |  |
| *Achyrocline satureioides* (Lam.) DC. | Marcela, macela, macelinha |
| *Baccharis articulata* (Lam.) Pers. | Carqueja-doce, carqueja, vassoura |
| *Baccharis crispa* Spreng. | Carqueja-verdadeira, carqueja-amarga, vassourinha |
| *Baccharis dracunculifolia* DC. | Vassourinha, alecrim-do-campo, alecrim-vassoura |
| *Bauhinia forficata* Link | Pata-de-vaca, casco-de-vaca, pata-de-boi, pata-de-touro, unha-de-vaca, unha-de-boi, mororó |
| *Bromelia antiacantha* Bertol. | Banana-do-mato, caraguatá, bananinha-de-macaco |
| *Casearia Sylvestris* Sw. | Chá-de-bugre, erva-de-bugre, guaçatonga |
| *Cecropia glaziovii* Snethl. | Embaúba, embaúva, embaúba-vermelha |
| *Copaifera trapezifolia* Hayne. | Pau-óleo, capaíba, copaibeira, capuva, óleo |
| *Croton celtidifolius* Baill. | Pau-sangue, sangue-de-dragão, tapicingui |
| *Cunila microcephala* Benth. | Poejo, poejinho, poejo-do-banhado |
| *Drimys brasiliensis* Miers | Casca-de-anta, cataia, para-tudo, canela-armaga, caátuya |
| *Echinodorus grandiflorus* (Charm. & Schltr.) Micheli | Chapéu-de-couro |
| *Equisetum giganteum* L. | Cavalinha, rabo-de-cavalo |
| *Hypericum caprifoliatum* Cham. & Schltdl | Orelha-de-gato, escadinha, sinapismo, hipérico |
| *Ilex paraguariensis* A.St.-Hil. | Erva-mate, chá-verde, |
| *Jodina rhombifolia* (Hook. & Arn.) Reissek | Cancorosa-de-três-pontas, iodina, jodina |
| *Mikania glomerata* Spreng. | Guaco, guaco-liso, cipó-caatinga, erva-de-cobra |
| *Mikania laevigata* Sch.Bip. ex Baker | Guaco cheiroso, guaco |
| *Mimosa scabrella* Benth | Bracatinga, abracatinga, bracatinho, paracatinga, anizeiro, mandengo |
| *Ocimum Carnosum* (Spreng.) Link & Otto ex Benth. | Alfavaca-anisada, alfavaca-cheiro-de-anis, elixir-paregórico, anis, alfavaquinha, alfavaca-preta, erva-das-mulheres |
| *Plantago australis* Lam. | Tansagem, tanchagem |
| *Piper umbellatum* L. | Pariparoba, pariparova, periparoba, capeba, caena, aguaxima, caapeba, catajé, lençol-de-santa-bárbara, jaguarandi |
| *Sambucus australis* Cham. & Schltdl. | Sabugueiro, sabugueiro-do-rio-grande, sabugueiro-do-brasil |
| *Smilax campestris* Griseb. | Salsaparrilha, sarsaparrilha, japecanga, japicanga, jupicanga, nhapecanga, zarza, ivapeca |
| *Solanum mauritianum* Scop. | Fumo-brabo, cuvitinga, couvetinga, tabaqueira, fona-de-porco, bugweed, wild-tobacco, tree-tobacco |
| *Solanum paniculatum* L. | Jurubeba, jurubeba-verdadeira, jurubebinha, jurupeba |
| *Sorocea bonplandii* (Baill.) W.C.Burger et al., Zollernia ilicifolia (Brongn.) Vogel, Jodona rhombifolia (Hook. & Arn.) Reissek | Falsas-espinheira-santas, espinheiras-santas |
| *Trichilia catigua* A.Juss. | Catiguá, cataguá, catuaba, amarelinho, aroeirinha, angelim-rosa, mangaltô-catinga, |
| *Varronia curassavica* Jacq. (Boraginaceae) | Erva-baleeira, salicina, balieira, maria-preta |
| *Wilbrandia ebracteata* Cogn. | Taiuiá |
| *Zollernia ilicifolia* (Brongn.) Vogel | Coração de negro, ipê boia, laranjeira brava, laranjeira da mata, laranjeira do mato, Maria preta, mocetaiba, mucitaiba, mucitaiba preta, orelha de onça, pau santo |
| **Ornamental species** |  |
| *Ananas bracteatus* (Lindi.) Schult. & Schult.f. | Ananás-ornamental, abacaxi-silvestre, abacaxi-vermelho, abacaxi-ornamental, abacaxi-do-pará, red-pineapple, wild-pineapple |
| *Aspilia montevidensis* (Spreng.) Kuntze | Mal-me-quer-do-campo, mal-me-quer-amarelo, margarida-do-campo, aspília |
| *Calliandra tweedii* Benth. | Topete-de-cardeal, cardeal, quebra-foice, sarandi, espinho-vermelho, maricá-vermelho, esponjinha, esponjinha-vermelha |
| *Cortaderia selloana* (Schult.) Asch. | Capim-dos-pampas, penacho, cana-tinga, palha-de-penacho, pluma-de-capim, bandeira, pluma, tiririca, macega, paina |
| *Dyckia distachya* Hassi. | Gravatá, bromélia |
| *Epidendrum fulgens* Brongn. | Orquídea-da-praia, epidendro, orquídea-de-restinga |
| *Fuchsia regia* (Vell.) Munz | Brinco-de-princesa |
| *Gomesa flexuosa* (Lodd.) M.W.Chase & N.H.Williams | Chuva-de-ouro, orquídea-dançarina |
| *Handroanthus chrysotrichus* (Mart. Ex DC.) Mattos | Ipê-amarelo, ipê-do-morro, ipê-amarelo-cascudo, ipê-tabaco |
| *Heliconia farinosa* Raddi | Caité, caité-banana, bananeirinha-do-mato, helicônia |
| *Jacaranda puberula* Cham. | Caroba, caroba-da-mata, carobeira, caroba-roxa, carobinha, jacarandá-branco |
| *Parodia ottonis* (Lehm.) N.P.Taylor | Tuna-de-bola, tuninha, mandacaruzinho, tuna-amarela, cactus-bola |
| *Petunia integrifolia* (Hook.) Schinz & Thell. | Petúnia-perene |
| *Pyrostegia venusta* (Ker Gawl.) Miers | Flor-de-são-joão, cipó-de-são-joão-amarelo |
| *Rumohra adiantiformis* (G.Forst.) Ching | Samambaia-preta, samambaia-silvestre, iron-fern, leather-leaf-fern, hojas-de-cuero |
| *Sinningia leucotricha* (Hoehne) H.E.Moore | Rainha-do-abismo |
| *Syagrus romanzoffiana* (Cham.) Glassman | Jerivá, gerivá, coqueiro-gerivá, coqueiro, coco-de-cachorro, baba-de-boi, coco-de-catarro, coco-de-babão |
| *Tibouchina sellowiana* Cogn. | Manacá-da-serra, manacá, quaresmeira, quaresmeira-da-serra, jacatirão |
| *Trichocline catharinensis* Cabrera | Cravo-amarelo-do-campo, cravo-amarelo |
| *Verbena rigida* Spreng | Erva-arame, camaradinha, verbena, sand-paper-verbena, vained-verbena, tuberous-vervain, stiff-vervain, veined-verbena |
| **Timber species** |  |
| *Apuleia leiocarpa* (Vogel) J. F. Macbr. | Grápia, guarapiapinha, grapiapunha, garapa. |
| *Aspidosperma polyneuron* Mull.Arg. | Peroba-rosa, peroba, peroba-açu |
| *Ateleia glazioveana* Baill. | Timbó, timbozinho, cinamomo-bravo |
| *Balfourodendron riedelianum* (Engl.) Engl. | Guatambu, pau-marfim, farinha-seca |
| *Cabralea canjerana* (Vell.) Mart. | Canjerana, canharana, cajarana |
| *Calophyllum brasiliense* Cambess. | Olandi, olandim, landi, landim |
| *Cedrela fissilis* Vell. | Cedro, cedro-batata, acaiacá |
| *Colubrina glandulosa* Perkins | Sobraji, sobrasil, sobraju |
| *Cordia trichotoma* (Vell.) Arráb ex Steud. | Louro-pardo, louro-batata, ajui |
| *Enterolobium contortisiliquum* (Vell.) Morong | Timbaúba, orelha-de-macaco, tamburé |
| *Handroanthus heptaphyllus* Mattos | Ipê-roxo, ipê-rosa, ipê-preto |
| *Hieronyma alchorneoides* Allemão | Licurana, aricurana, abacateiro |
| *Miconia cinnamomifolia* (DC.) Naudin | Jacatirão-açu, jacatirão, carvalho-vermelho |
| *Mimosa scabrella* Benth. | Bracatinga, abracatinga, anizeiro |
| *Nectandra lanceolata* Nees | Canela-amarela, canela-branca, canela-louro |
| *Ocotea puberula* (Rich.) Nees | Canela-guaicá, amansa-besta, louro-pimenta |
| *Parapiptadenia rigida* (Benth.) Brenan | Angico, angico-amarelo, angico-cedro |
| *Peltophorum dubium* (Spreng.) Taub. | Canafistula, cássia-amarela, faveira |
| *Piptocarpha angustifolia* Dusén ex Malme | Vassorão-branco, vassourão |
| *Schizolobium parahyba* (Vel.) S. F. Blake | Guapuruvu, ficheira, pataqueira |

Table S4. Most relevant bioclimatic variables selected *a priori* based on different plant growth forms.

| **Plant Growth Forms** | ***a priori*** | **References** |
| --- | --- | --- |
| Epiphyte (n=2) | bio1, bio2, bio4, bio6, bio7, bio10, bio12, bio14, bio15, bio18, bio19 | ^1–3^ |
| Fern (n=1) | bio1, bio3, bio4, bio7, bio8, bio9, bio10, bio11, bio13, bio18, bio19 | ^4,5^ |
| Graminoid (n=27) | bio1, bio2, bio4, bio5, bio8, bio10, bio11, bio12, bio15, bio16 | ^6–8^ |
| Herb (n=26) | bio1, bio3, bio4, bio5, bio7, bio8, bio9, bio12, bio13, bio14 | ^9,10^ |
| Hydrophyte (n=2) | bio3, bio4, bio6, bio7, bio8, bio10, bio11, bio12, bio14, bio15, bio18, bio19 | ^11–13^ |
| Lithophyte (n=1) | bio1, bio4, bio7, bio12, bio15 | ^14^ |
| Shrub (n=17) | bio2, bio4, bio5, bio6, bio8, bio9, bio16, bio17, bio18 | ^15,16^ |
| Tree (n=53) | bio1, bio3, bio4, bio5, bio6, bio11, bio12, bio14, bio15, bio16, bio17 | ^17,18^ |
| Vine (n=10) | bio1, bio2, bio6, bio7, bio12, bio14, bio17 | ^19,20^ |

Table S5. Variance inflation factor values (VIFs) inspected to different plant growth forms.

| Variables | IUCN Plant Growth Forms Classification Scheme | | | | | | | | |  |
| --- | --- | --- | --- | --- | --- | --- | --- | --- | --- | --- |
|  | Epiphyte  (n=2) | Fern  (n=1) | Graminoid  (n=27) | Herb  (n=26) | Hydrophyte  (n=2) | Lithophyte  (n=1) | Shrub  (n=17) | Tree  (n=53) | Vine  (n=10) | |
| Annual Mean Temperature (bio1) | 1.861835 | - | - | - | - | 1.679218 | - | - | 1.896634 | |
| Mean Diurnal Range (bio2) | 2.167821 | - | 1.922928 | - | - | - | 1.795278 | - | 3.230660 | |
| Isothermality (bio3) | - | 2.809173 | - | 3.117781 | 3.482705 | - | - | 1.909455 | - | |
| Temperature Seasonality (bio4) | 2.723942 | - | 2.826974 | - | - | - | 2.448608 | - | - | |
| Max Temperature of Warmest Month (bio5) | - | - | - | - | - | - | 1.294166 | 1.296326 | - | |
| Temperature Annual Range (bio7) | - | 3.113471 | - | 4.033197 | 3.411926 | 2.089461 | - | - | 4.313900 | |
| Mean Temperature of Wettest Quarter (bio8) | - | - | - | 2.746230 | - | - | - | - |  | |
| Mean Temperature of Driest Quarter (bio9) | - | - | - | 4.663764 | - | - | - | - |  | |
| Mean Temperature of Warmest Quarter (bio10) | - | 1.401655 | 1.334304 | - | 1.288196 | - | - | - |  | |
| Annual Precipitation (bio12) | - | - | - | - | - | 2.307550 | - | - | 3.844531 | |
| Precipitation of Wettest Month (bio13) | - | 4.170311 | - | 2.436467 | - | - | - | - | - | |
| Precipitation of driest month (bio14) | 3.925511 | - | - | 1.415324 | 3.729690 | - | - | 2.692639 | 2.574091 | |
| Precipitation Seasonality (bio15) | 2.471926 | - | 1.563038 | - | 2.222454 | 1.226550 | - | 2.154748 | - | |
| Precipitation of Wettest Quarter (bio16) | - | - | 2.675161 | - | - | - | 3.400874 | 2.321465 | - | |
| Precipitation of Driest Quarter (bio17) | - | - | - | - | - | - | 2.338722 | - | - | |
| Precipitation of Warmest Quarter (bio18) | 2.173990 | 1.575626 | - | - | 1.851238 | - | 2.047170 | - | - | |
| Precipitation of Coldest Quarter (bio19) | 2.191400 | 2.219854 | - | - | 2.138370 | - | - | - | - | |

Table S6. ODMAP (Overview, Data, Model, Assessment, Prediction) protocol for ecological niche models

Climate change threatens Brazilian agroforestry plant species

– ODMAP Protocol –

Authors: -

2021-06-01

## Overview

#### Authorship

Contact: [-](mailto:vallldeir@gmail.com)

#### Model objective

Model objective: Forecast and transfer

Target output: Continuous habitat suitability and binary maps of potential presence for each species

#### Focal Taxon

Focal Taxon: Brazilian agroforestry native plant species

#### Location

Location: Brazil, mainly areas covering the Atlantic Forest and Pampa grasslands

#### Scale of Analysis

Spatial extent (lon/lat): 65^o^ W - 33^o^ W, 3^o^ S - 34^o^ S

Temporal extent/time period: Current data for 1970-2000 and future for 2041-2060 and 2061-2080

Boundary: Political

#### Biodiversity data

Observation type: field survey

Response data type: presence-only

#### Predictors

Predictor types: climatic

#### Hypotheses

Hypotheses: Species BAHs are affected by climate change. We quantify potential changes in BAH for 139 native agroforestry species from the Brazilian flora using two different climate change scenarios (SSP2-4.5 and SSP5-8.5) for 2041-2060 and for 2061-2080.

#### Assumptions

We assume that (1) the species are at equilibrium with the environment, (2) have stable niches, (3) occurrence records sampled randomly, (4) predictors are free of errors and (5) habitat suitability as a proxy of species BAH.

#### Algorithms

Modelling techniques: MaxEnt v.3.4.1k

Model complexity: We only kept linear and quadratic features to avoid overfitting of the models and as recommended by Merow *et al.* because of the absence of a biological justification with the variables used ^21,22^.

#### Workflow

Current and future potential habitats for species were predicted using MaxEnt v.3.4.1k. To assess robustness and alert policy-makers for the uncertainties typically associated with these methods, each ENM was tested against a bias corrected null-model as proposed by Raes and ter Steege ^23^. The climate projections were carried out according to the Sixth Assessment Report (AR6) of the IPCC, using two Shared Socioeconomic Pathways (SSPs) as reference (SSP2-4.5 and SSP5-8.5). We averaged eight different global climate models: BCC-CSM2-MR, CNRM-CM6-1, CNRM-ESM2-1, CanESM5, IPSL-CM6A-LR, MIROC-ES2L, MIROC6 and MRI-ESM2-0 to take into account the uncertainties related to future climate conditions ^24^.

#### Software

All modelling and tests were carried out within the R environment ^25^, using the R packages ‘dismo’ ^35^, ‘flora’ ^33^, ‘rgdal’ ^27^, ‘rgeos’ ^28^, ‘rJava’ ^29^, ‘raster’ ^31^, ‘sf’ ^32^ and ‘usdm’ ^30^.

Code availability: Available on request

Data availability: Datasets generated are available from GBIF: <https://doi.org/10.15468/dl.vjezvb>

## Data

#### Biodiversity data

Taxon names: Taxon names are listed in the Supplementary Information.

Taxonomic reference system: We standardized botanical names using the R package ‘flora’, which uses the nomenclature accepted by the Brazilian Flora 2020 project (<http://floradobrasil.jbrj.gov.br/>).

Ecological level: species

Data sources: All species occurrence data was obtained from the online database of the Global Biodiversity Information Facility (<https://doi.org/10.15468/dl.vjezvb>)

Sampling design: We extracted all records from the following extent -90, -25, -70, 20 to avoid modelling truncated niches

Sample size: The sample size for species ranged from 12 (*Ornithopus micrantus*) to 5464 (*Casearia sylvestis*).

Background data: We drew 10,000 points of background for each species’ BAH independently.

#### Data partitioning

#### Model performance was assessed using a bias corrected null-model as proposed by Raes and ter Steege.

#### Predictor variables

Annual Mean Temperature (bio1), Mean Diurnal Range (bio2), Isothermality (bio3), Temperature Seasonality (bio4), Max Temperature of Warmest Month (bio5), Temperature Annual Range (bio7), Mean Temperature of Wettest Quarter (bio8), Mean Temperature of Driest Quarter (bio9), Mean Temperature of Warmest Quarter (bio10), Annual Precipitation (bio12), Precipitation of Wettest Month (bio13), Precipitation of driest month (bio14), Precipitation Seasonality (bio15), Precipitation of Wettest Quarter (bio16), Precipitation of Driest Quarter (bio17), Precipitation of Warmest Quarter (bio18) and Precipitation of Coldest Quarter (bio19)

Data sources: Worldclim version 2.1. (<http://worldclim.org>)

Spatial extent: -90, -25, -70, 20

Spatial resolution: The raw resolution of the climate data was 5 arc-minutes

Coordinate reference system: WGS 1984

Temporal extent: Temporal extent of raw data was 1970-2000

#### Transfer data

Data sources: Worldclim version 2.1. (<https://www.worldclim.org/data/cmip6/cmip6climate.html>)

Spatial extent: -90, -25, -70, 20

Spatial resolution: The raw resolution of the future climate data was 5 arc-minutes

Temporal extent: 2041-2060 and 2061-2080

Models and scenarios: We averaged eight different global climate models: BCC-CSM2-MR, CNRM-CM6-1, CNRM-ESM2-1, CanESM5, IPSL-CM6A-LR, MIROC-ES2L, MIROC6 and MRI-ESM2-0

Quantification of Novelty: As transferring models across time can lead to spurious extrapolations and consequently unrealistic predicted ENMs, we adopted a conservative approach by fitting the models to the estimated species BAHs.

## Model

#### Multicollinearity

Multicollinearity: We checked for multicollinearity by examining the correlation structure of the predictor variables through the variance inflation factor (VIF). We kept only predictors with VIF values below 5 ^103^. The VIFs were checked using the function ‘vifstep’ in the R package ‘usdm’ ^104^.

#### Model settings

ENMs were fitted using the following parameters in the MaxEnt: bootstrap method with 100 replicates, 500 maximum iterations, 10,000 points of background, and Cloglog output format. We only kept linear and quadratic features to avoid overfitting of the models and as recommended by Merow *et al.* because of the absence of a biological justification with the variables used ^21,22^.

#### Model estimates

We computed variable importance of evaluation runs.

#### Analysis and Correction of non-independence

None

#### Threshold selection

To map changes in future ranges of species, we converted the continuous habitat suitability into binaries using the maximum training sensitivity plus specificity threshold ^78,113^.

## Assessment

#### Performance statistics

#### Model performance was assessed using a bias corrected null-model as proposed by Raes and ter Steege.

#### Plausibility check

Response shapes: we inspected species-response-curves to avoid spurious calibrations, following the evaluation strip method proposed by Elith *et al*.^112^.

## Prediction

#### Prediction output

Prediction unit: Continuous habitat suitability and binary maps of potential presence for each species.

#### Uncertainty quantification

Scenario uncertainty: For future climate change predictions, we averaged eight global climate change models.

Figure S1. Potential native agroforestry plant species changing from a current assessed IUCN category or Not Evaluated (NE) to a threat category, Vulnerable (VU), Endangered (EN) and Critically Endangered (CR), based on different major uses due to climate change.


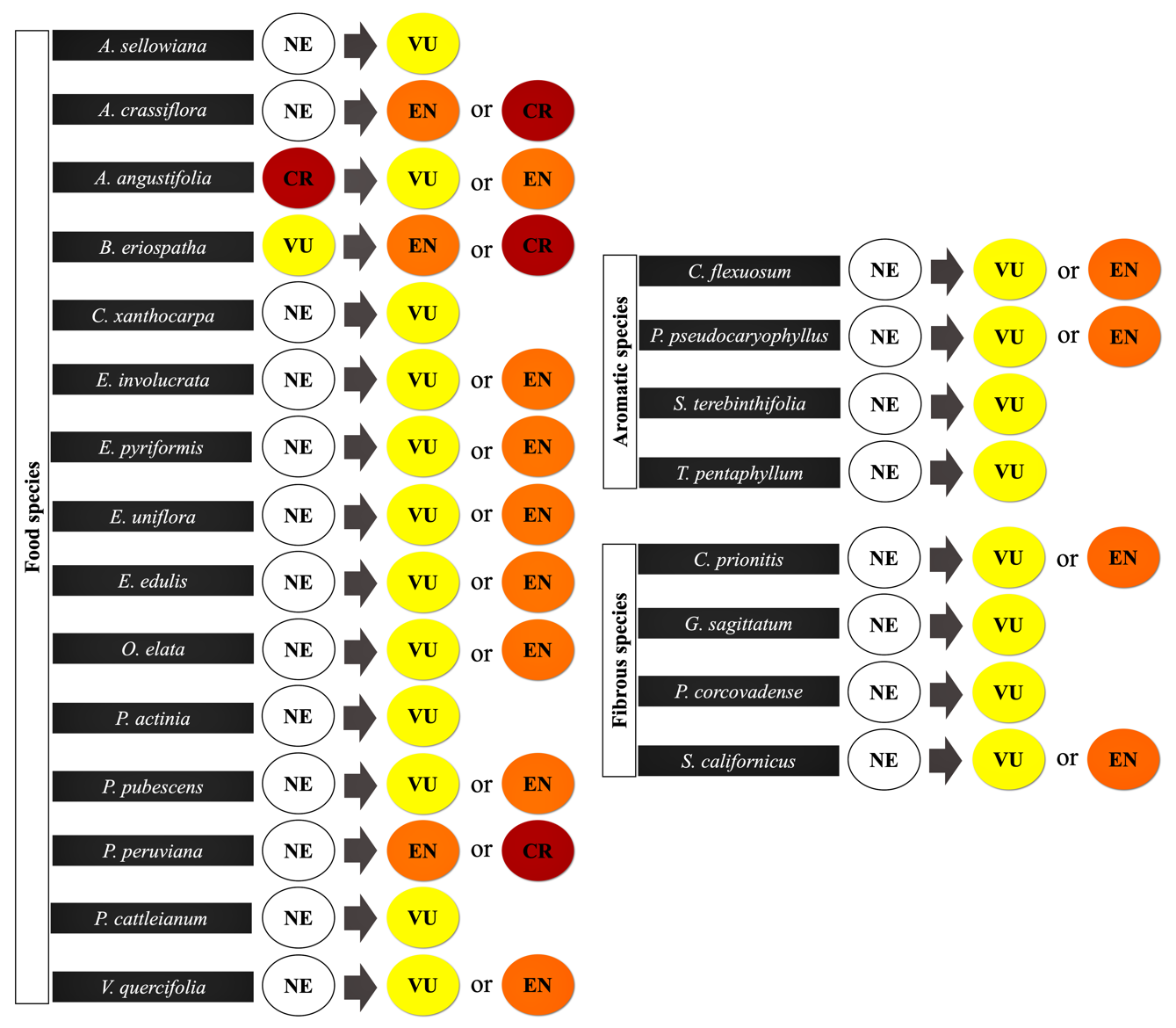


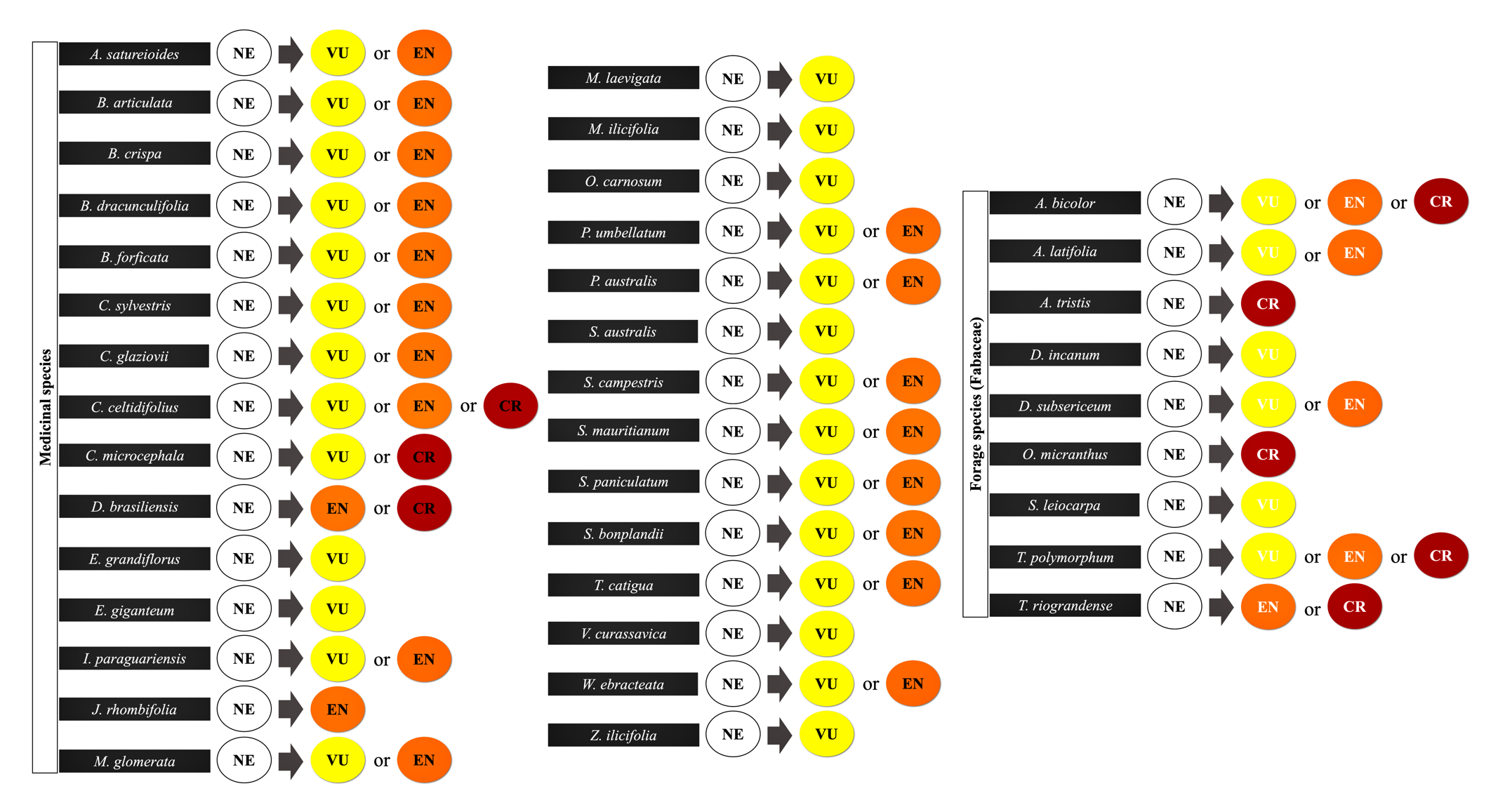


**References**

1. Aguiar-Melo, C. *et al.* Ecological niche modeling and a lack of phylogeographic structure in Vriesea incurvata suggest historically stable areas in the southern Atlantic Forest. *Am. J. Bot.* (2019) doi:10.1002/ajb2.1317.

2. Kolanowska, M., Rewicz, A. & Baranow, P. Ecological niche modeling of the pantropical orchid Polystachya concreta (Orchidaceae) and its response to climate change. *Sci. Rep.* (2020) doi:10.1038/s41598-020-71732-1.

3. Maciel, J. R., Sánchez-Tapia, A., De Siqueira, M. F. & Alves, M. Palaeodistribution of epiphytic bromeliads points to past connections between the Atlantic and Amazon forests. *Bot. J. Linn. Soc.* (2017) doi:10.1093/botlinnean/bow020.

4. de Gasper, A. L., Eisenlohr, P. V. & Salino, A. Climate-related variables and geographic distance affect fern species composition across a vegetation gradient in a shrinking hotspot. *Plant Ecol. Divers.* **8**, 25–35 (2015).

5. de Gasper, A. L., Grittz, G. S., Russi, C. H., Schwartz, C. E. & Rodrigues, A. V. Expected impacts of climate change on tree ferns distribution and diversity patterns in subtropical Atlantic Forest. *bioRxiv* (2020) doi:https://doi.org/10.1101/2020.01.16.909614.

6. Cupertino-Eisenlohr, M. A. *et al.* Stability or breakdown under climate change? A key group of woody bamboos will find suitable areas in its richness center. *Biodivers. Conserv.* (2017) doi:10.1007/s10531-017-1332-x.

7. Albuquerque, F., Macías-Rodríguez, M. Á., Búrquez, A. & Rowe, H. Toward an understanding of broad-scale patterns of the habitat suitability of fountain grass (Cenchrus setaceus (Forssk.) Morrone, Poaceae). *Plant Ecol.* **221**, 1029–1043 (2020).

8. Karunarathne, P., Feduzka, C. & Hojsgaard, D. Ecological setup, ploidy diversity, and reproductive biology of Paspalum modestum, a promising wetland forage grass from south america. *Genet. Mol. Biol.* (2020) doi:10.1590/1678-4685-gmb-2019-0101.

9. Vieira, L. T. A. *et al.* Geographical patterns of terrestrial herbs: a new component in planning the conservation of the Brazilian Atlantic Forest. *Biodivers. Conserv.* (2015) doi:10.1007/s10531-015-0967-8.

10. Barros, M. J. F. *et al.* Environmental drivers of diversity in Subtropical Highland Grasslands. *Perspect. Plant Ecol. Evol. Syst.* (2015) doi:10.1016/j.ppees.2015.08.001.

11. Lehtonen, S. On the origin of echinodorus grandiflorus (Alismataceae) in Florida (‘E. Floridanus’), and its estimated potential as an invasive species. *Hydrobiologia* (2009) doi:10.1007/s10750-009-9903-9.

12. Alahuhta, J., Heino, J. & Luoto, M. Climate change and the future distributions of aquatic macrophytes across boreal catchments. *J. Biogeogr.* **38**, 383–393 (2011).

13. Heneidy, S. Z., Halmy, M. W. A., Fakhry, A. M. & El-Makawy, A. M. The status and potential distribution of Hydrocotyle umbellata L. and Salvinia auriculata Aubl. under climate change scenarios. *Aquat. Ecol.* (2019) doi:10.1007/s10452-019-09705-4.

14. Melo, E. A. & Waechter, J. L. Beta diversity patterns of bromeliaceae growing on rocky cliffs within the atlantic forest in Southern Brazil. *Biota Neotrop.* (2020) doi:10.1590/1676-0611-BN-2019-0846.

15. Amaral, A. G., Munhoz, C. B. R., Walter, B. M. T., Aguirre-Gutiérrez, J. & Raes, N. Richness pattern and phytogeography of the Cerrado herb–shrub flora and implications for conservation. *J. Veg. Sci.* **28**, 848–858 (2017).

16. Silva, K. J. P. & Souza, A. F. Common species distribution and environmental determinants in South American coastal plains. *Ecosphere* (2018) doi:10.1002/ecs2.2224.

17. Gomes, V. H. F., Vieira, I. C. G., Salomão, R. P. & ter Steege, H. Amazonian tree species threatened by deforestation and climate change. *Nat. Clim. Chang.* **9**, 547–553 (2019).

18. Zwiener, V. P. *et al.* Planning for conservation and restoration under climate and land use change in the Brazilian Atlantic Forest. *Divers. Distrib.* **23**, 955–966 (2017).

19. Teixeira, M. C., Mäder, G., Silva-Arias, G. A., Bonatto, S. L. & Freitas, L. B. Effects of past climate on Passiflora actinia (Passifloraceae) populations and insights into future species management in the Brazilian Atlantic forest. *Bot. J. Linn. Soc.* (2016) doi:10.1111/boj.12375.

20. Barros, M. J. F., Diniz-Filho, J. A. F. & Freitas, L. B. Ecological drivers of plant genetic diversity at the southern edge of geographical distributions: Forestal vines in a temperate region. *Genet. Mol. Biol.* **41**, 318–326 (2018).

21. Merow, C., Smith, M. J. & Silander, J. A. A practical guide to MaxEnt for modeling species’ distributions: What it does, and why inputs and settings matter. *Ecography.* **36**, 1058–1069 (2013).

22. Boucher-Lalonde, V., Morin, A. & Currie, D. J. How are tree species distributed in climatic space? A simple and general pattern. *Glob. Ecol. Biogeogr.* **21**, 1157–1166 (2012).

23. Raes, N. & ter Steege, H. A null-model for significance testing of presence-only species distribution models. *Ecography.* **30**, 727–736 (2007).

24. Araujo, M. & New, M. Ensemble forecasting of species distributions. *Trends Ecol. Evol.* **22**, 42–47 (2007).

25. R Core Team. R: A language and environment for statistical computing. R Foundation for Statistical Computing, Vienna, Austria. (2020).

26. Thuiller, W., Lafourcade, B., Engler, R. & Araújo, M. B. BIOMOD - A platform for ensemble forecasting of species distributions. *Ecography.* (2009) doi:10.1111/j.1600-0587.2008.05742.x.

27. Bivand, R., Keitt, T. & Rowlingson, B. Package ‘rgdal’. *R Packag.* (2016) doi:10.1353/lib.0.0050.

28. Bivand, R. & Rundel, C. rgeos: Interface to Geometry Engine - Open Source ('GEOS’). R package version 0.5-2. (2019).

29. Urbanek, S. rJava: Low-Level R to Java Interface. R package version 0.9-11. (2019).

30. Naimi, B. Package ‘ usdm ’. *R Topics Document* (2015).

31. Hijmans, R. J. raster: Geographic Data Analysis and Modeling. R package version 3.0-2. (2019).

32. Pebesma, E. Simple features for R: Standardized support for spatial vector data. *R J.* (2018) doi:10.32614/rj-2018-009.

33. Carvalho, G. flora: Tools for Interacting with the Brazilian Flora 2020. R package version 0.3.0. (2017).

34. Valavi, R., Elith, J., Lahoz‐Monfort, J. J. & Guillera‐Arroita, G. blockCV: An r package for generating spatially or environmentally separated folds for k-fold cross-validation of species distribution models. *Methods Ecol. Evol.* **10**, 225–232 (2019).

35. Hijmans, R. J., Phillips, S., Leathwick, J. & Elith, J. Package ‘dismo’’ - Species Distribution Modeling’. *CRAN Repository* (2017).

36. Phillips, S. J. *et al.* Sample selection bias and presence-only distribution models: implications for background and pseudo-absence data. *Ecol. Appl.* **19**, 181–197 (2009).

37. Barbet-Massin, M., Jiguet, F., Albert, C. H. & Thuiller, W. Selecting pseudo-absences for species distribution models: How, where and how many? *Methods Ecol. Evol.* (2012) doi:10.1111/j.2041-210X.2011.00172.x.

38. Elith, J. *et al.* Novel methods improve prediction of species’ distributions from occurrence data. *Ecography.* **29**, 129–151 (2006).

39. Roberts, D. R. *et al.* Cross-validation strategies for data with temporal, spatial, hierarchical, or phylogenetic structure. *Ecography.* **40**, 913–929 (2017).

40. Xiao-Ge, X., Tong-Wen, W. & Jie, Z. Introduction of CMIP5 Experiments Carried out with the Climate System Models of Beijing Climate Center. *Adv. Clim. Chang. Res.* **4**, 41–49 (2013).

41. Yeager, S., Karspeck, A., Danabasoglu, G., Tribbia, J. & Teng, H. A Decadal Prediction Case Study: Late Twentieth-Century North Atlantic Ocean Heat Content. *J. Clim.* **25**, 5173–5189 (2012).

42. Jones, C. D. *et al.* The HadGEM2-ES implementation of CMIP5 centennial simulations. *Geosci. Model Dev.* **4**, 543–570 (2011).

43. Watanabe, S. *et al.* MIROC-ESM 2010: model description and basic results of CMIP5-20c3m experiments. *Geosci. Model Dev.* **4**, 845–872 (2011).

44. Watanabe, M. *et al.* Improved Climate Simulation by MIROC5: Mean States, Variability, and Climate Sensitivity. *J. Clim.* **23**, 6312–6335 (2010).

45. Fremout, T. *et al.* Mapping tree species vulnerability to multiple threats as a guide to restoration and conservation of tropical dry forests. *Glob. Chang. Biol.* **26**, 3552–3568 (2020).

46. Bean, W. T., Stafford, R. & Brashares, J. S. The effects of small sample size and sample bias on threshold selection and accuracy assessment of species distribution models. *Ecography.* **35**, 250–258 (2012).

47. Jiménez-Valverde, A. & Lobo, J. M. Threshold criteria for conversion of probability of species presence to either–or presence–absence. *Acta Oecologica* **31**, 361–369 (2007).

48. Elith, J., Ferrier, S., Huettmann, F. & Leathwick, J. The evaluation strip: A new and robust method for plotting predicted responses from species distribution models. *Ecol. Modell.* **186**, 280–289 (2005).
